# Comparison of the central human and mouse platelet signaling cascade by systems biological analysis

**DOI:** 10.1186/s12864-020-07215-4

**Published:** 2020-12-22

**Authors:** Johannes Balkenhol, Kristin V. Kaltdorf, Elmina Mammadova-Bach, Attila Braun, Bernhard Nieswandt, Marcus Dittrich, Thomas Dandekar

**Affiliations:** 1 Functional Genomics and Systems Biology Group, Department of Bioinformatics, Biocenter, Am Hubland, University of Würzburg, D-97074 Würzburg, Germany; 2 Institute of Experimental Biomedicine, University Hospital and Rudolf Virchow Centre, University of Würzburg, Würzburg, Germany; 3 Present address: Division of Nephrology, Department of Medicine IV, Hospital of the Ludwig, Maximilian University of Munich, D-80336 Munich, Germany; 4 Member of the German Center for Lung Research (DZL), Walther-Straub-Institute for Pharmacology and Toxicology, Ludwig-Maximilians University Munich, Munich, Germany; 5 Dept of Genetics, Biocenter, Am Hubland, University of Würzburg, Am Hubland, D 97074 Würzburg, Germany

**Keywords:** Interspecies comparison, Transcriptome, Proteome, Platelet, Network, Signaling, Mouse, Human, Interactome, Cascade

## Abstract

**Background:**

Understanding the molecular mechanisms of platelet activation and aggregation is of high interest for basic and clinical hemostasis and thrombosis research. The central platelet protein interaction network is involved in major responses to exogenous factors. This is defined by systemsbiological pathway analysis as the central regulating signaling cascade of platelets (CC).

**Results:**

The CC is systematically compared here between mouse and human and major differences were found. Genetic differences were analysed comparing orthologous human and mouse genes. We next analyzed different expression levels of mRNAs. Considering 4 mouse and 7 human high-quality proteome data sets, we identified then those major mRNA expression differences (81%) which were supported by proteome data. CC is conserved regarding genetic completeness, but we observed major differences in mRNA and protein levels between both species. Looking at central interactors, human PLCB2, MMP9, BDNF, ITPR3 and SLC25A6 (always Entrez notation) show absence in all murine datasets. CC interactors GNG12, PRKCE and ADCY9 occur only in mice. Looking at the common proteins, TLN1, CALM3, PRKCB, APP, SOD2 and TIMP1 are higher abundant in human, whereas RASGRP2, ITGB2, MYL9, EIF4EBP1, ADAM17, ARRB2, CD9 and ZYX are higher abundant in mouse. Pivotal kinase SRC shows different regulation on mRNA and protein level as well as ADP receptor P2RY12.

**Conclusions:**

Our results highlight species-specific differences in platelet signaling and points of specific fine-tuning in human platelets as well as murine-specific signaling differences.

**Supplementary Information:**

The online version contains supplementary material available at 10.1186/s12864-020-07215-4.

## Summary

The signal network of the central regulatory cascade in platelets was reconstructed. Transcriptomics and proteomics data of specific expression differences between human and mouse platelets were compared for this central cascade.

## Background

Blood platelets are anucleated small cells released from megakaryocytes (MKs) of the bone marrow into the blood. Circulating platelets adhere and aggregate at sites of vascular injury and together with the coagulation system form a fibrin rich clot to arrest bleeding [1]. On the other hand, platelets can cause pathological thrombosis and vessel occlusion leading to the most common life-threating pathologies, myocardial infarction and stroke [2, 3] and are involved in many other (patho) physiological processes, such as tissue healing, fibrosis, inflammation, angiogenesis and tumor metastasis [4–8]. The platelet protein and molecule interaction network involved in response to those exogenous factors is defined by systems biological pathway analysis as the central regulating signaling cascade of platelets (CC). The networks are composed of activatory and inhibitory up- and downstream pathways, involves major platelet proteins and mediates a fine-tuned balance of equilibrated blood flow [9]. The mapping of the CC is highly instructive for a better understanding of how platelet pathways are regulated in pathophysiological conditions.

Signaling cascades such as this important one implied in stroke, heart attack and cardiovascular disease in general [1–3] are often studied in model organisms such as the mouse. However, the many differences between the model genome and transcriptome and the human counter-part are rarely taken into account by the research groups studying the specifics of such a cascade [4–8]. Moreover, a global approach for our example, the platelet signaling cascade [9–11], was never attempted and was also not possible as critical data-sets for such a comparison were hard to come by. We can only provide an eagle’s perspective as testing each difference found by our systematic systems biological comparison in detail would be a new, time-demanding individual experimental study. We thus present here the first and thorough analysis of this signaling cascade of the platelet showing exactly where the genome biology and protein expression differs between mouse and man. We verify meticulously the differences observed by multiple data-sets comparing genome, transcriptome and proteome and give insight on the resulting functional implications considering latest data and available literature so that the genome biology differences of model organisms for central signaling cascades will no longer be ignored, at the very least for our chosen example.

Methods of bioinformatics have already been used to simulate basic signaling mechanisms regulating platelet aggregation [9–11]. Thereby, data sets of several knock-out and knock-in mouse models [12–14] have been useful to validate data. Differences between mouse and human signaling cascades have been observed in several cell types including platelets [15–17]. Therefore, systematic analyses of mouse and human platelet signaling cascades are required to estimate limitations of transferability of generated results and stress human specifics including potential therapeutic targets. Therefore, extensive transcriptome and proteome datasets and the latest genome updates are curated and compared here. We used the best available bioinformatics tools for systematic analyses to validate genetic differences between mouse and human. We included orthology analysis for interspecies comparison, accurate RNA expression and detailed evaluation of supporting or contradicting proteome evidence. Confirmed species-specific differences are discussed here in the context of their effects on the central signaling cascade.

## Results

Using a recently published model of the central activating cascade of the platelet (CC; includes inhibition by cAMP) [9], we systematically compared the most important signaling cascades described in both human and mouse platelets. For this study, we have used integrated genomic data followed by complete ortholog mapping of transcriptome and proteome datasets to compare the CC between mouse and human, using only correctly corresponding proteins and genes (orthology) and test their expression levels using platelet RNA and protein datasets. All available platelet transcriptome data were used to screen and evaluate potential differences between human and mouse. For meticulous validation, we used eleven recent high-quality proteome and phosphoproteome datasets [18–28] and compared them (detailed information in Supplemental Material and Methods). To consider only validated protein-protein interactions, we mapped our large-scale genome/transcriptome and proteome datasets using a reconstructed protein interactome of mouse and human platelets (see methods and Table S1 where full protein names are given). We next considered all available further large-scale and specific experimental data to validate differences between mouse and human (Table S2; full names included). In 46% of the cases, we could confirm RNA expression differences by similar protein expression differences after normalization (Table S2). In further 35% of the cases, the evidence was only compatible with the prediction, the available information from the proteome was not conflicting with the observed RNA difference (Table S2). It is important to note that in 19% of the cases proteome and RNA expression data showed clear opposite differences between the species (Table S2), suggesting different regulation on RNA or protein level and requiring further experimental investigation. This concerned four proteins (SRC, TBXA2R, PTGDR, RASGRP1) in the central cascade, as well as 8/37 1st neighbors plus 4/38 2nd degree neighbors (proteins explained in Supplemental file 1, data in Table S2; 99 mRNA differences investigated).

**Fig. 1:**
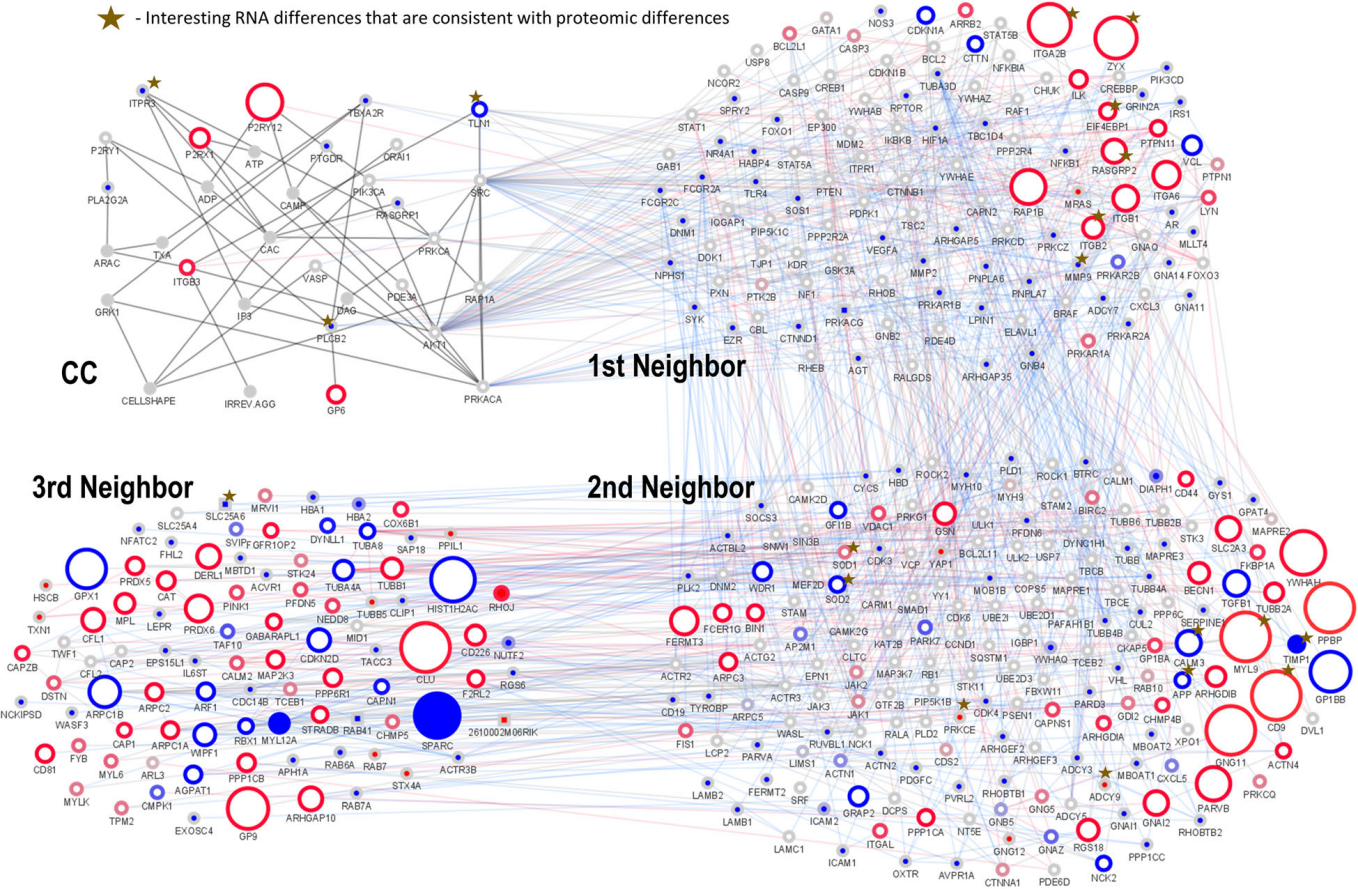
Differences in the central regulatory cascade (CC) between mouse and human. The center of the human and murine signaling cascade (defined according to systems biological modelling) and its regulators are presented in a combined network including proteomic, transcriptomic, metabolic and ionic interactors (full data Fig. 2). In thick edges, the main regulatory interactions are highlighted. The neighbors up to degree 3 are presented (see methods overview for an exact definition of 1st to 3rd degree neighbors of the **CC**. Asterisks label confirmed key expression differences of platelet proteins between human and mouse. As the platelet transcript and validated protein content is around 10,181 (9811 protein-coding) in human and 5981 (5814 protein-coding) in mice, large interaction networks can be reconstructed (Human: 18618 high confident interactions and 3524 interactors, Mouse: 10337 high confident interactions and 2114 interactors). In order to outline the important direct and indirect regulators of the central cascade that mark a difference in both species, the combined network shows solely the clear differences (filtered) of a subset of the global interaction network from the first to third neighbors of the central cascade (full: 1811 nodes and 11,527 edges; filtered: 411 nodes and 1959 edges). The combined central network separated into species results in 1618 nodes and 9406 edges in human (Fig. S2), as well as 1061 nodes and 5769 edges in mice (Fig. S3). The filtered combined central network results in 369 nodes and 1646 edges in human, as well as 277 nodes and 1119 edges in mice. The first to the third neighbor network was filtered according to clear genomic or transcriptomic differences (interspecies expression differences > 100 RPKM; expressed > 10 RPKM in one species whereas not in the other; no ortholog found between species according to Inparanoid^8^; connector between those proteins). The human and mouse network were combined. The differences in both network topologies are shown in color code. The border paint marks expression values (blue for high expression in human; red for high expression in mouse; grey for no evident expression differences). The node paint marks proteins that occur only in human platelet network (blue), only in human (blue rectangle; non-ortholog proteins), only in murine platelet network (red), only in mouse (red rectangle; non-ortholog proteins), or in both (white). The grey fill color of nodes indicates proteins that are not expressed in platelets in either species. Second messengers (e.g. Calcium, ATP, ADP) are also shown in grey. The node size increases with high expression differences. Edge colors indicate interactions in both species (grey), in human (blue), in mouse (red) and in the central cascade (dark grey). Selected high protein expression differences which are shown by transcriptomics and proteomics accordingly (Table 1 and Fig. 3) are highlighted by golden asterisks. High binders above 90% percentile were excluded. Abbreviations in the figure are the Entrez gene symbols and the full names are given for all genes in Supplemental Table 1. Supplemental Fig. 4 is a separate png file and a high-resolution version of Fig. 1. It allows to inspect better individual subnetworks around different proteins, in particular around interesting species differences (see asterisks in the figure) and the corresponding protein and gene expression differences between species

**Fig. 2:**
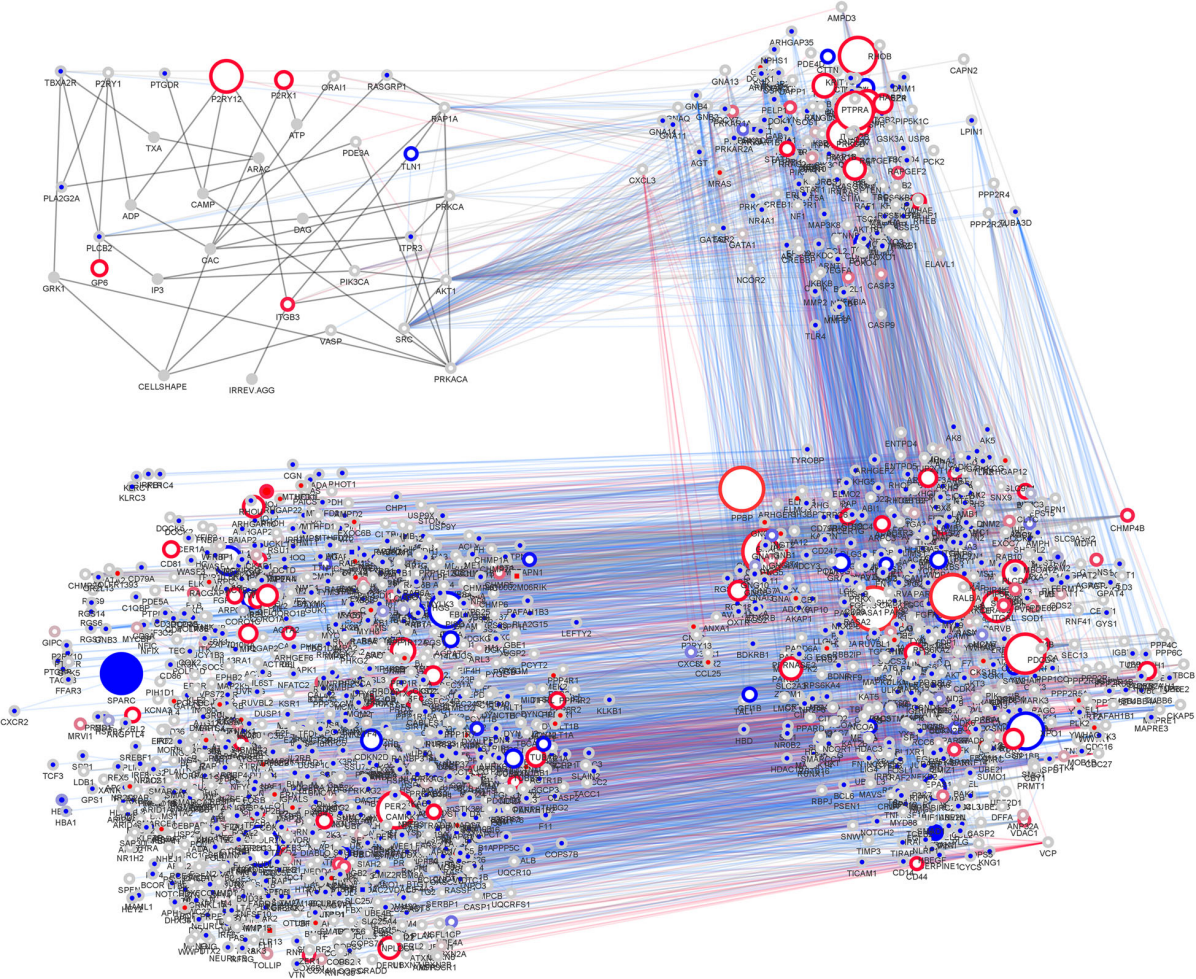
Full Network of proteins in and around the central platelet signaling cascade. The human and mouse networks were combined. The differences in both network topologies are shown in color code. The border paint marks expression values (blue for high expression in human; red for high expression in mouse; grey for no considerable expression differences). The node paint marks proteins that occur only in human platelet network (blue), only in human (blue rectangle; non-ortholog proteins), only in murine platelet network (red), only in mouse (red rectangle; non-ortholog proteins), or in both (white). The grey fill color of nodes indicates proteins that are not expressed in platelets in both species, or second messenger (e.g. Calcium, ATP, ADP). The node size increases with high expression differences. Further, edge color indicates interactions in both species (grey), in human (blue), in mouse (red) and in the central cascade (dark grey). High binders above 90% percentile where excluded. Abbreviations in the figure are the Entrez gene symbols and the full names are given for all proteins in Supplemental Table 1. Supplemental Fig. 5 is a separate jpeg file and a high-resolution version of Fig. 2. It allows to inspect better individual networks around different proteins, and the corresponding protein and gene expression differences between species

In the next analysis step, we focused on all confirmed expression differences. The combined data compared the same proteins in mouse and human (direct seed orthologs) to reveal differences (Fig. 1; detailed full network in Fig. 2). In total, 1132 proteins were confirmed to have the same function in both species (all are direct orthologs). Table 1 lists the species networks for human and mouse. 621 human mRNAs are solely contained in the human network and 58 murine mRNAs are only found in the murine network. Besides species-specific variation in proteins found in human and mouse platelets, this results in species-specific subnetworks including differences for well-connected orthologs (same protein in mouse and human with more or less or sometimes different connections depending on species). The proteins in the networks are represented according to their mRNA evidence.

**Table 1:** Key expression differences in the central platelet signalling cascade

gene symbol/full name	cascade position^*^	experimental data
**higher abundant in human**		
TLN1 Talin 1	central, increased in human	[15, 18–28]
CALM3 Calmodulin 3	2nd neighbour^*^, increased in human	[15, 18, 21–23, 26, 27]
PRKCB Protkinase C^a^	2nd neighbour, strong increase in human	[15, 18–25, 27]
APP amyloid ßA4^b^	2nd neighbour, clearly increase in human	[15, 18–25]
SOD2 SuperoxidDis^c^	2nd neighbour, clearly increase in human	[15, 18–21, 23–25, 27]
TIMP1 Protease inhib^d^	2nd neighbour, clearly only human, T	[15, 18, 20–22, 24]
**Only Human**		
PLCB2 Phospholipase C^e^	central, only human, good expressed)	[15, 18–22, 24]
MMP9 Metalloprotease^f^	1st degree^*^, only human, low expressed	[15, 20]
BDNF brain derived factor^g^	2nd degree^*^,	[15, 18, 20, 22]
ITPR3 triphosphat receptor^h^	central, low expression	[15, 18]
SLC25A6 Solute carrier^i^	3rd degree^*^, low expressed	[15, 18, 20–24]
**higher abundant in mouse**		
RASGRP2 Guanyl release^j^	1st neighbour	[15, 18–27]
ITGB2 Integrin Beta 2	1st neighbour, good difference,	[15, 20, 25–27]
MYL9 myosin regulation^k^	2nd neighbour, high expression/ difference	[15, 18, 20–27]
EIF4EBP1 initiation factor^l^	1st neighbour, very clear difference	[15, 20, 25, 26]
ADAM17 metallopeptidase^m^	3rd neighbour, clear difference	[15, 18, 19, 25, 26]
ARRB2 Arrestin ß2	1st neighbour, good difference	[15, 18, 20, 25, 26]
CD9 Complement^n^	2nd neighbour, good marker, higher in mouse	[15, 18, 20–25, 27]
SOD1 SuperoxidDis^c^	2nd neighbour, high abundant in both	[15, 18–21, 24–27]
ZYX Zyxin protein	1st neighbour, high expression in both	[15, 18–28]
**Only Mouse**		
GNG12 Guanin binding^o^	2nd degree, high expression, clear difference	[15, 25–27]
PRKCE protein kinase Cε	2nd degree, high expression, clear difference	[15, 25, 26]
ADCY9 Adenylate Cyclase 9	2nd degree, high expression, clear difference	[15, 25, 26]
**Divergent expression levels comparing RNA versus protein**		
**Glycoprotein VIb** (only form present in platelets)		
For GP6b (glycoprotein VIb (platelet) the mRNA level tends to be increased in mice, but the protein level shows clearly higher abundance in human (mouse higher in transcriptome whereas human higher in proteome)		
**SRC / Src protein kinase** shows different mRNA and protein level regulation in man and mouse (human higher in transcriptome whereas mouse higher in proteome); the higher proteome expression has a clear effect on the switching behaviour of SRC as bistability switch (13)		

*Neighbor definition: see methods overview; 2nd degree = 2nd degree neighbor; 3rd degree = 3rd degree neighbor

**Abbreviations:** ^a^*Protkinase C* Protein kinase C, isoform B, ^b^*amyloid ßA4* amyloid beta A4 percursor protein, ^c^*SuperoxidDis* superoxide dismutase, ^d^ *Protease inhib* TiM metallopeptidase inhibitor 1, ^e^*Phospholipase C* Phospholipase C, isoform B2, ^f^*Metalloprotease* Matrixmetalloprotease 9, ^g^*BDNF* Brain derived neurotrophic factor, ^h^*ITPR3* inositol 1,4,5 triphosphat receptor type 3, ^i^*SLC25A6* Solute carrier family 25 (mitochondrial) member 6, ^j^*RASGRP2* RAS-Guanyl releasing protein 2, ^k^*myosin regulation* myosin light polypeptide 9, ^l^*EIF4EBP1* eukaryotic translation initiation factor 4E binding protein1, ^m^*metallopeptidase* ADAM metallopeptidase domain 17, ^n^*CD9 antigen* Complement defining protein 9, ^o^*Guanin binding* Guanin nucleotide binding protein gamma 12

Using the similarity of conserved pathways the combined network supports the network reconstruction of each species. The current reconstructed network of *human* platelets encompasses 1608 proteins and 9406 interactions (Fig. S2). The *murine* network comprises of 1051 proteins and 5769 interactions (Fig. S3). The direct comparison of each species network covers 858 direct ortholog proteins and 3648 shared interactions. The combined network (Fig. 2) has 1801 proteins. Half of these proteins (903) are abundant in platelets in at least one of the two species (RPKM > 3; adjusted threshold according to the median of the central cascade).

**Fig. 3:**
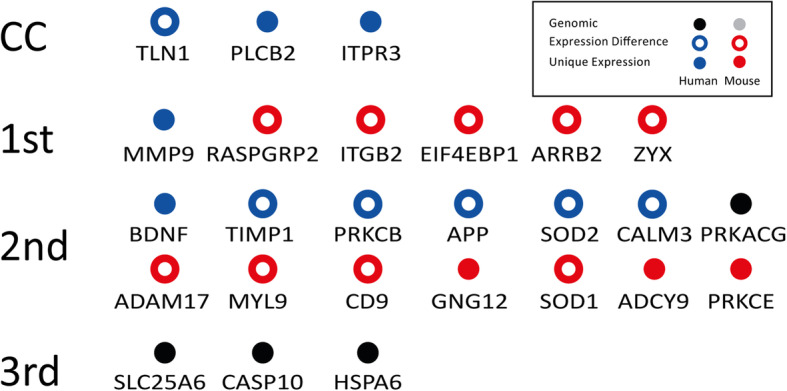
Overview of the key expression differences between mouse and human platelet CC. Simplified overview on the found differences for the platelet CC: (i) The key set of proteins that have clear expression differences between mouse and human in the CC or its neighbors as confirmed by transcriptomics and proteomics data are shown (blue rings: higher in human, red rings: higher in mouse). Genetic differences are shown as black points (gene found only in human; for the mouse no such clear difference was found). Moreover, we found cases where there was only expression found in one of the species (“unique”) though in both species the gene was present (blue dot: unique in human; red dot: unique in mouse)

Key results (asterisk) of this comparison between mouse and human are indicated in Fig. 1 and summarized in Fig. 3, individual differences are discussed in Supplemental Material taking all available proteome and RNA datasets into account.

### Overall expression and network differences

The overview of the central regulatory proteins and the central cascade shows that murine proteins involved in platelet signaling are expressed at higher levels (median RPKM: 4.5) compared to human platelets (median RPKM: 2). The cumulative expression (RPKM) in mouse was also much higher (total RPKM: 96420) compared to human (total RPKM: 53487). We found that the well-studied human signaling network includes a higher number of proteins (1608) compared to model organism mouse (1051). In the human network up to degree 3, we identified 33 proteins with a relatively high RPKM (over 100). In contrast, within the mouse signaling network, 82 proteins were detected with high RPKM (more than 100). The full central network with all regulators up to neighbor degree 3 results in 1618 nodes and 9406 edges in human, and 1061 nodes and 5769 edges in mouse (including non-protein interactors). Our calculation included also the signaling molecules which belong to the CC, according to Mischnik et al., [9] Although the CC is assumed to be conserved between mouse and human species, using all available information from databases and experiments, we found a number of clear genetic differences as well as different mRNA and protein levels in mouse and human platelets. Figure 1 shows the resulting network (asterisks label key differences), the CC and its neighbors, including 369 nodes and 1646 edges in human and 277 nodes and 1119 edges in mouse.

In addition, we also compared the total platelet network of mouse and human (Fig. 2). The human network contains 3524 nodes with 18,618 high confidence protein interactions (almost certainty; *p* > 0.99). The average number of protein interactions was about 5 interactors per signaling protein. In comparison, a high confidence dataset in the IntAct database [29] reports 9 interactors per protein and only 6 interactors by excluding high binders. In sharp contrast, the complexity of the mouse network was found to be reduced, only 2114 nodes and 10,337 interactions were identified. Nevertheless, similar network properties were found and the average number of interactors was 5 per protein. The overall analysis presented here has no species bias using a homogenous prediction method. All major differences found for the CC, its direct neightbors and 2nd or 3rd degree neighbors are concisely summarized in Fig. 3.

### Specific differences in the central cascade

The systems biological defined CC [9–11] showed no genomic difference between human and mouse platelets (Fig. 3). However, abundance differences of mRNA and protein could be identified in the CC (Fig. 1; blue borders indicate higher expression in human and red indicates higher expression in mouse; proteins directly interacting with the CC are 1st degree neighbors of the CC, interactors of these are 2nd degree neighbors and the proteins interacting only with the 2nd degree neighbors in turn are 3rd degree neighbors). PLCB2 (phospholipase C beta 2) and ITPR3 (inositol 1,4,5-triphosphate receptor type 3) have not been detected in mouse on mRNA level, but are expressed in human (matches proteome evidence). Talin (TLN1) mRNA is higher abundant in human which is confirmed by proteomics (Table S2).

RNAseq and proteome datasets could not provide firm evidence for the detection of relevant expression levels of Phospholipase A2 Group IIA (PLA2G2A) in both species in transcriptome, as well as proteome. TBXA2R (thromboxane A2 receptor) shows a higher protein level in mouse but the absence of mRNA in mouse and high mRNA expression in human. PTGDR (prostaglandin D2 receptor (DP)) only has mRNA expression in human and no protein evidence was found in both species. The same is valid for RAS guanyl-releasing protein 1 (RASGRP1). Purinergic receptor signaling is regulated by P2RY12 (purinergic receptor P2Y, G-protein coupled, 12) and P2RX1 (purinergic receptor P2X, ligand-gated ion channel 1). mRNA expression levels of these receptors, which are directly activated by ADP and ATP, respectively, [30, 31] are clearly higher in mouse. In accordance with this, there is clear protein quantification of P2RY12 receptor in murine platelets (log2: 1.3; Zeiler et al., 2014 [25] and 2.0 according to Hurtado et al., 2018 [26]) but P2RY12 protein in human is low and difficult to detect (Table S2). It is present in really low and variable amounts [32] but easy measured as functionally present receptor [33]. These concordant results of mRNA and proteome support a difference in central receptor signaling between mouse (higher expression of P2RY12) and human. For the calcium channel *P2RX1* and the collagen receptor Gp6 (GPVI in human platelet) higher mRNA expression in mouse was found but proteome data suggest opposite protein abundance. *ITGB3* (integrin beta 3) differs slightly on mRNA level, but not on protein level. In addition, the highly expressed central platelet signaling kinase SRC [34] shows clear differences, although mRNA and protein level give opposite estimates suggesting independent regulation.

### Major mouse-human platelet proteome expression differences

Key proteome differences of the CC are summarized in Table 1. There are clear differences in the regulation and modulation of the central cascade between man and mouse. In particular, sometimes a protein counter part in the other organism is lacking or almost absent, there are strong expression differences. Each of these clear differences with functional implications for the platelet has been several times reported and observed in literature (Table 1).

Higher abundance of copper-zinc-superoxide dismutase 1 (SOD1) in murine platelets implies better ROS protection [35]. In human platelets, manganese-dependent superoxide dismutase 2 (SOD2 in the mitochondrial matrix) is higher abundant (Table S2). It regulates apoptotic pathways and expression differences influence also platelet apoptosis-like activation [36].

The talin abundance difference is important as it regulates key proteins in platelets [9] such as integrin influencing thrombosis and platelet adhesion [37]. In particular, Talin decreases integrin activation and reduces the probability of the platelet for irreversible aggregation [38].

Glycoprotein VI (GP6 or GPVI), the platelet receptor for collagen, laminin and fibrin, centrally regulates multiple platelet functions, including adhesion, activation, aggregation and pro-coagulant activity [39–44].

Matrix metalloproteinases (MMPs) are reorganizing the extracellular matrix [45, 46]. MMP9 is only present in humans, its low expression affects platelet activation [47] (Table S2 and [48]). Platelet expression differences in tissue inhibitors of metalloproteinase (TIMPs), such as TIMP1, TIMP2 and TIMP3 affect activities of MMPs and by this platelet aggregation [49, 50].

Brain-derived neurotrophic factor (BDNF) is described only in humans. Protective effects for brain [51] are mediated by platelet BDNF and impaired by smoking [52, 53]. There are gender differences as well as BDNF expression differences in patients with cardiovascular disease and depression. All these brain protective effects mediated by platelet BDNF are absent in mouse platelets, there is no similar protein (ortholog) present. Src protein kinase shows opposite differences regarding mRNA versus protein level regulation in man and mouse (Table S2). This implies that Src kinase as the central bistability switch of the activating cascade [10] has different activation tipping points in man and mouse.

More detailed functional relevance of proteins pointed out in the paper or found to be different in the CC network are given in the supplementary material.

### Specific genomic differences in the 1st to 3rd degree neighbors

Five human genes not detected in the mouse genome are 1st to 3rd degree neighbors of the central cascade: SLC25A6, CASP10, PRKACG, HSPA6 and RAB41. Details are given in Table S2 and the Supplemental data file considering all available data-sets.

### Different expression profile of the 1st to 3rd degree neighbors

We looked at proteins directly interacting with the central cascade (1st neighbors of CC) or interactors of these (2nd neighbors of CC) or one interaction further (3rd degree neighbors of CC) using well established human and murine interaction data. A first screen analyzed mRNA expression differences in both species after normalization, next a detailed comparison according to support or lack of support in the eleven large-scale platelet proteome studies was done (see materials and methods for details including log2 value calculations and comparison protocols).

According to this census of all available data, the following further differences were found for direct interacting protein neighbors (1st neighbors) of the central cascade analyzing their differential expression:

There are 44 proteins, which are not detected in mouse, but identified in human platelets. Within this group, PRKAR1B (log2: 4.7 (mRNA) and 0.7 (protein)), IRS1 (log2: 2.8 (mRNA) and − 0.4 (protein)), DNM1 (log2: 2.3 (mRNA) and 2.7 (protein)) and FCGR2A (log2: 1.7 (mRNA) and 0.5 (protein)) are the most relevant. HABP4 has only mRNA but no protein expression in human (log2: 1.5 for mRNA; no detection in any of the proteome studies), thus suggesting the expression of a non-coding RNA (ncRNA); XR_001746249; miscRNA). MRAS and KDR are clearly detected as murine mRNA (log2: 3.2 and log2: − 0.6) but lack protein evidence in both species. Similar, mouse mRNAs of DOCK1, NF1 and TJP1 were only detected in mouse but protein levels are unclear, or in the opposite expression difference to the mRNA expression level differences. DOCK1 is slightly expressed on protein level with a small tendency to be increased in murine mRNA and protein. NF1 and TJP1 levels are low when present, indicating the proximity to detection sensitivity limits. A higher mRNA expression level of four proteins, namely VCL, CDKN1A, CTTN and protein kinase cAMP-dependent type II regulatory subunit beta (PRKAR2B) was found in human platelets. Proteome datasets supported these differences for VCL and CDKN1A, although the extent is lower on protein levels. For CTTN high abundance levels are detected in transcriptomics, as well as in proteomics for mouse and human, with a trend for higher levels in humans. PRKAR2B is similarly confirmed to be an abundant protein in both species, but shows clearly higher protein level in mouse.

In mouse platelets, 17 mRNAs were found to be expressed higher than in human platelets, such as Integrin αIIβ, RAP1B (matching several proteome data-sets), ITGA6 (proteome data find the opposite species difference to the RNA data), ITGB1 (compatible), RASGRP2, ITGB2, ZYX, ILK (matches proteome data), EIF4EBP1, PTPN11 (matches), LYN (matches), ARRB2, PRKAR1A (compatible), BCL2L1 (compatible), CASP3 (matches), PTPN1 (matches), PTK2B (matches) and STIM1 (matches proteome; full information and names are given in Table S1, S2). RASGRP2, ITGB2, EIF4EBP1, ARRB2, and ZYX are particularly interesting candidates for further investigation with substantial mRNA differences that are in accordance with proteomic differences.

Regarding the 2nd neighbors (543 human mRNAs) we identified 134 proteins in human such as TIMP1 which were not found in mouse platelets. There are 46 direct ortholog mRNAs that are clearly higher abundant in mice such as CD9 and SLC2A3 though also detected in human (details in Supplementary Data).

Referring to mRNA levels, in the 3rd degree neighbor network, 285 mRNAs are only detected in human, but not in mouse platelets, whereas 58 mRNAs are exclusively detected in mouse platelets. 24 of the 3rd neighbor mRNAs show a stronger expression in human. 46 platelet mRNAs are stronger expressed in mice, e.g. CLU and RHOJ (details in Supplementary Data).

## Discussion

This study starts from a transcriptome-based search for differences in protein expression between mouse and human central platelet cascade followed by extensive validation of the found potential differences by eleven recent high-quality proteome datasets [18–28]. Hence we have here a solid comparative basis to discuss and evaluate the differences in the CC of platelet regulation between human and mouse focussing only on those that are confirmed considering all these data. By this, we filter differences most likely to be relevant for high network control of platelets.

We found in 19% of the data evidence for opposite regulation at transcriptome and proteome level, which suggests alternative regulation of both, such as genomic regulation, or post-translational modification [54–57]. However, for the majority of proteins (81%), their comparative mRNA abundance in one organism is accompanied by matching protein abundance: this could be valid also for other organisms and related comparative studies [54–57]. We focus here on such cases but mention also unclear cases or cases where the expression differences on RNA level are confirmed to be in the opposite direction to the protein level differences. Differences between transcriptome and proteome abundancies may arise during platelet biogenesis.

Due to the large data sets (see Table S1), we highlight in the paper only important species-specific differences, particular those where mRNA and protein expression differences are in accordance. There are many more protein and RNA expression differences between mouse and human platelets in the neighbor proteins; we discuss all confirmed differences in detail in the Supplemental Material.

In total, we analyzed 1811 expression differences in the combined network. Besides genetic differences between human and mouse, we followed in detail those major mRNA expression differences which were strongly supported by proteome data considering 4 mouse and 7 human proteome data sets [18–28] and from the further 19% of the cases only those with a clearly opposite regulation between proteome and mRNA level.

We used data sets containing four mouse and seven human proteomes. This is a small series, however, these were all available public large-scale studies with suitable data. Nevertheless, we stress the statistical limitations of the study with this comparatively small n. Hence, for the key differences summarized in Table 1, we give supporting references. For the gene expression the figures become more solid as many reads are compared to each other in each study (higher n). For the proteome the internal standards in each study allow estimating for the higher expressed proteins a higher security for the observed differences. Although Table 1 gives some additional references for the found functional differences, specific differences should be followed up by targeted experiments to further investigate their significance.

The platelet central cascade is genetically conserved between human and mouse, but there are some expression differences. A surprising amount of differences become apparent considering its 1st to 3rd degree neighbors which critically modulate platelet responses. These differences, including expression of central activating receptors, metalloproteases, SOD and smaller differences in the cytoskeleton network are currently under further investigation. Our analysis of these omics data is fully made available here, but it is of course no substitute for direct functional tests, which have to follow up all of these differences and sometimes did already so in the past.

A global approach evaluating the platelet signaling cascade was never attempted and not possible due to a lack of sufficient data. Related studies in the field include the seminal work comparing the transcriptome of mouse and human of the Weyrich group [15], In fact, this study triggered our endeavour, we added more information on RNA expression from the PlateletWeb systems biology workbench [58] and its updates, but then systematically compared these results to the available large-scale proteomics data-sets on platelets, focussing and investigating here the CC of the platelet and its neighbors. A method-related study by Uosaki and Taguchi [16] concerns instead exclusively microarray data-sets and does a comparison of mouse and human gene expression profiles in cardiac maturation. Moreover, there is a study by Schmidt et al. [59] which revealed by comparative proteomics a number of quantitative phosphorylation differences linked to platelet activation state looking exclusively at human platelets. The authors compared the human non-secretory platelet proteome, considering both in-vitro activation and inhibition to platelet controls in 2D gel electrophoresis.

We hence take such comparisons one clear step further regarding the platelet CC and provide here for the first time a systematic overview (eagle’s view) on all differences found between human and mouse proteome and transcriptome and the reader can testify there are a lot of differences. We combine here high-quality proteome [18–28] and transcriptome data sets [15, 58]. High-quality data refers here to studies that are well published, described, reviewed with good data access and in particular could be correctly mapped on reference genes. Shortcomings and biases, such as limited transcriptome studies or proteome studies with only cytosplamic data [28], or a lack of parametric and non-parametric correlation due to their high data diversity, or allowing no complete mapping of the data to the two reference genomes were excluded from the census. All information on recent large-scale platelet proteomics studies (since 2010) was considered. Data were again tested on their coherence with PlateletWeb database [58] and its references on individual transcriptome and proteome observations.

Wherever possible, we provide also literature and data on tests of specific differences, in particular for all major differences and their functional relevance and impact as listed in Table 1. However, please note that testing each difference found by our systematic systems biological comparison in further detail would be a new, time-demanding individual experimental study. It is very clear from this comparison that the term model organism of course implies that details of the signaling cascade are different, in particular regarding expression. This becomes obvious and numerous if several large-scale data-sets for the platelet CC are compared. With even more data our analysis will become more complete. Furthermore, we found cases where the expression difference on the RNA level is opposite to the difference found for the protein level. This is partially explained by the limitations of the data-sets (ambiguous case), but for several cases this is clearly an indication for translational regulation further modulating the differences between RNA and protein level including support from literature [54–57].

This is the first global analysis of the platelet CC. However, the expression differences and genetic differences of model organisms for central signaling cascades should generally better be taken into account, taking this study on the central signaling cascade of the platelet as a blueprint.

Taken together, the differences given in Table 1 imply that overall the CC behaves similar in mouse and human. However, the detailed regulation differs with specific implications for individual differences as outlined above. In particular for therapeutic studies, long-term effects, chronic disease conditions as well as rapid activation of the CC it is clear that any helpful observation and insight from the mouse models has to be closely validated for human platelets as details and modulation are often somewhat different. Hence without validation in human platelets, a step by step transfer into clinical treatment is not possible. However, as these spots of differences in expression, modulation, and interaction are all assembled here together regarding the CC, this typical challenge can now be mastered more efficiently for the platelet CC.

## Conclusion

We document variation between human and mouse regarding the expression of proteins and mRNA for the central cascade of platelet activation and fine-tuned modulation including its interactors. Genetic differences occur only in 1st to 3rd degree interactors. As the central cascade is genetically well-conserved, the mouse represents a good model for platelet (patho-)physiology while transfer to clinic and patients including drug development requires to take the shown differences closely into account. Our data will help to improve the design of future studies, point out some limitations of the mouse model and provide detailed data to interpret the regulatory mechanisms of platelet activation, including drug targeting, regulation of hemostasis, thrombosis, thrombo-inflammation and cancer. The study serves as a blueprint for similar comparisons in other signaling cascades.

## Methods

This study aims to compare the central platelet signaling network in platelets of mouse and human. For this the platelet protein interaction networks were reconstructed based on a comparative RNAseq dataset [15]. In the reconstructed network relevant pathways were curated and filtered by bioinformatics analysis [10, 11], focusing on the central regulating cascade of platelet activation according to systems biological modelling (CC), the directly CC interacting proteins (1st degree neighbors) as well as the neighbor proteins (2nd degree neighbor of the CC) and finally, the neighbors of these 2nd degree neighbors (3rd degree neighbors of the CC). The differences in the central pathways were closely investigated and further analyzed by proteomics. For this, recent proteome datasets were collected, integrated and normalized. Thus, differences in central pathways were outlined by genomics, transcriptomics and followed up by proteomics. The flow-chart (Fig. S1) shows how the different methods explained in the following were consecutively applied.

### Platelet network reconstruction in mouse and human

For our protein-protein interaction screening of platelets, available data were used to determine presence or absence of genes coding for the proteins in mouse and or human, which are part of the central response cascade of the platelet (CC) as defined by systems biological modelling of key platelet responses [9–11]. Therefore, the human platelet proteome was compared to the mouse platelet proteome [60] and sequence orthology (i.e. verification of whether two sequences are describing the same proteins with the same function in both species) was determined by using the Inparanoid software [61]. Blast version 2.2.26 was run with the following parameters to build orthologous groups: scoring matrix Blosum62, a score-cut-off of 40 bits, a sequence overlap of 0.5, a group merging cut-off of 0.5 and a minimum score of 0.05. Non-orthologous proteins were also identified by these parameters.

To meticulously compare, normalize and score the datasets on proteins and protein interactions, we took into account all available high-quality experimentally validated data, large-scale proteomics datasets and interaction data sets of the platelet. Details on the protein and interaction curation are given in the supplement. The analysis of platelet protein expression started from using the PlateletWeb knowledge base [58] but considered available latest data and platelet proteome updates, in particular, the datasets [62–67] regarding the CC (up to 3rd-degree neighbors) and the analysis was applied to both RNA and protein data. Furthermore, platelet transcriptome data (RNA, NGS data), such as described by Rowley et al., [15], was incorporated for mouse and human. Data [9] on molecules and drugs associated with the central cascade were added. Important for identifying neighbors in protein-protein networks are the conncetions. The protein-protein connections were retrieved from experimentally verified data sets. We considered only all experimentally verified and validated interactions, but combined such predictions from IntAct [29] and BioGrid [68]. Another challenge is to compare the resulting networks from mouse and man as there are local differences. Hence, first all homologus proteins between man and mouse were identified. Next, all interactions were mapped onto a joined network interconnecting all orthologous proteins (those with same domain composition and same function) from mouse and man as well as all non-orthologous proteins (so the mouse-specific or human-specific protein were added, too). Finally, from this combined network (Fig. 2) then the human-specific (Fig. S2) and the mouse-specific network were derived (Fig. S3). Hence, a prediction method [69, 70] for setting up the conserved network topology and proteins between man and mouse was established to which then the human-specific or mouse-specific proteins were specifically added in their respective network. In the next section we describe the curation of this network.

### Curating platelet protein interactions and comparing mouse and human

The detailed steps for curation of the protein annotation, interaction information and the mapping of the expression information (see methods flow diagram) are described as follows: The interactions of seven model species including different kingdoms (*H. sapiens, M. musculus, D. rerio, C. elegans, S. cervisiea, A. thaliana, E. coli*) were combined for a basic integrated network of evolutionary relevant interactions. From those known interactions actual interactions in human and mice are deduced via a protein sequence similarity framework using Inparanoid [61]. A Bayesian scoring method was applied following constraints, such as components directing the prediction power of systematic exchange of essential genes between human and yeast [71] and other [72–75]. We considered sequence similarity (global and local), sequences length, expression level, shared pathways, GO similarity, interacting domain similarity, quality of source interaction, evolutionary conservation (coevolution) and centrality of interaction.

The central cascade was determined following the central cascade given in the experimentally validated model described by Mischnik et al., [9]. Mapping this on the refined network, interacting neighbors up to degree 3 were annotated. The following input nodes from second messengers were included also in the network topology but are not further considered below as we focus exclusively on the proteins: CAC (cytosolic calcium), ATP, ADP, cAMP, DAG (diaglycerol), IP3 (inositoltriphosphat), ARAC (arachidonic acid), TXA2 (thromboxane A2). The murine and human network were combined in an overall network with differences indicated by color and size code referring on a transcriptomic difference between mouse and human. For this purpose the RPKM (reads per kilobase of exon model per million mapped reads) differences of comparative RNAseq experiments were used [15]. Expression differences across species could be reproduced for individual selected proteins by PCR analysis [15, 76]. The visualisation of the network was performed using Cytoscape 3.4.0 [77] software. For limiting the size of the network and the search space, bordering conditions had to be defined. Only clear differences were considered further in the combined network of human and mouse. These were (i) cross-species RPKM difference > 100 (was set as threshold to compare both networks to approximate a quantile above 90% of all differences); or (ii) proteins that are expressed solely in one of the two organisms (at least expressed > 10 RPKM, solidly over detection limit); (iii) clear genomic presence against absence in the other genome. The RPKM delta for the proteins was calculated by subtracting the murine RPKM from the human RPKM. Figure 1 (overview) shows protein expression. If there are RNA expression data available this is given in addition. Furthermore, the protein evidence in human platelets is more solid and better sampled than in mouse. This may contribute to an overestimation of the differences in protein content. The full network is available in Supplemental Table S1 (including the full names of all proteins) and displayed in Fig. 2.

### Normalization of RNAseq data in mouse and human data

We used the full genome annotation of mouse and human to identify proteins. Moreover, for direct comparison, all datasets were normalized. Regarding transcriptome data, starting with the data from the Weyrich group [15] with two independent isolation approaches, the RPKM (Reads per kilobase per million mapped reads) values of platelets were furthermore carefully normalized by comparing equal cell counts (~ 2 × 10^9^) [15]. In order to compare mRNA [15] and proteome data [18–28], we determined the median of the different semi-quantitative abundances for each study. The mRNA, respectively protein abundance relative to the median of each dataset was determined and transformed into a log2 scale (see Supplemental material and methods).

### Potential biases and supporting proteome evidence

Mouse platelets are considerably smaller than human platelets (MPV 4.7 vs. 7.5–10 fl) and the murine platelet counts of more than 30 strains results in a mean platelet count of ~ 1.1 × 10^6^ μL^− 1^ [78]. The normal platelet count in human ranges from ~ 150 × 10^9^/L to 400 × 10^9^/L (~ 0.15 × 10^6^ μL^− 1^ to ~ 0.4 × 10^6^ μL^− 1^) [79]. Moreover, human platelets have been studied for decades, whereas the mouse has been developed into a model organism only during the last 20–25 years. After this initial screen, we included all available data avoiding misrepresentations in the transcriptomic data. This includes 11 independent high-quality and large-scale platelet proteome studies [18–28], as well as further published evidence, e.g. [80, 81] and all data from our own repository at http://plateletweb.bioapps.biozentrum.uni-wuerzburg.de. Of note, Nygaard et al., [28] analyzed only the platelet cytosolic proteome. Further mouse studies were not considered as their data seemed not to be sufficiently complete for our comparison. Supplemental Table 2 summarizes the independent evidence for determined clear differences between human and mouse from our data, stressing where confirmatory evidence to the RNA expression differences is there from current proteome studies and where not.

### Normalization of proteomic data for comparing to RNAseq and between species

We used the available full genome annotation of mouse and human. Moreover, for direct data-set comparison all data-sets were normalized. Regarding transcriptome data, starting with the data from the Weyrich group [15] with two independent isolation approaches, the RPKM values of platelets were furthermore carefully normalized by taking in account equal cell counts (~ 1–3 × 10^9^) [15]. Despite being smaller in volume and with a higher density in blood the sum of averaged RPKM in mice is higher (789,877.7) than in human (388,576.8), reflecting higher expression levels in mice while in human the transcript diversity is higher. The study used (Weyrich) directly compared both transcriptomes. However, these gene expression data sets provided only a first suggestion for potential differences, we used then 11 recent proteome datasets to validate any observed differences [18–21, 23–28]. To compare these proteome data, we normalized by the median of the different semi-quantitative protein abundances as measured (measurements considered peptide spectrum count (PSM), protein copy number, ion abundance, and ion intensity). The log2fold change of protein abundance according to the overall median is stated and compared between the different proteome studies and in consequence to the log2fold change according to the median of RPKM data. By this approach, the differences between mouse and human achieve a common comparable platform. Table S2 depicts the log2fold changes of the different platelet RNA and the high-quality protein expression studies compared to RNA expression data from Rowley et al., 2011 [15], Burkhart et al., 2012 [18], Beck et al., 2016 [19], Sabrkhany et al., 2018 [20], Trugilho et al., 2017 [21], Solari et al., 2016 [22], Zufferey et al., 2014 [23], Rijkers et al., 2017 [24], Zeiler et al., 2014 [25], Hurtado et al., 2018 [26], Malmstrom et al., 2017 [27] and Nygaard et al., 2017 [28]; in addition individual proteins and RNA data from PlateletWeb [58] were considered. A color code indicates a higher or lower expression in the results figure. Log relative abundance expression levels are compared to log relative abundance levels of proteome studies. Due to a lack of data density (e.g. Nygaard et al., measured only cytosolic proteins), we selected for further analysis only the maximum expression value within a species. We scored clear differences of absence or presence of a mRNA and protein between mouse and human with 1 if confirmed by proteome, − 1 if proteome and transcriptome are opposed and 0 when no clear statement can be placed. For mutual occurring proteins, the tendency that e.g. a human protein is higher expressed than the murine gene was scored with 1 when confirmed by proteome and − 1 if not. A score of 0 was given when there was either no confirmed nor an opposed tendency. The third estimate analyzes the delta of log2fold changes between human and mouse. The similarity of delta was scored between 1 and − 1, from similar to not similar. All 3 scores are summed to an overall score for each protein. Due to different treatment of clear differences and delta differences the overall score ranges from − 2 to 2. Considering 99 proteins in Table S2, the summed overall score ranges from − 198 to 198. We state here a summed overall score of 58 for mRNA difference compared by proteome difference.

Correlation between proteome and transcriptome is only clear for about half of the proteins analyzed and we focus on those in the results section. The other half of the cases analyzed lacks such a clear picture. This can be due to objective differences in regulation [54–57] resulting from platelet biology such as translational regulation (mRNA would then stay constant while protein level in this organism is triggered upon stimulation) or due to technical limitations of the measurements, e.g. challenges in the isolation of membrane proteins in proteomics.

We considered score values greater or equal to 1 as matched, i.e. similar protein and mRNA differences were found between mouse and human. A score of less or equal to − 1 shows instead clear opposite results regarding organism-specific differences looking at the protein or the mRNA data set and was hence termed “opposite”. Though this may have good biological reasons, we focus in the results text only on those examples, where the biological reason for such a divergence and biological regulation was evident from publications.

Finally, the score between − 1 and 1 was termed “compatible”, if at least one proteome comparison confirmed and supported the mRNA measurements regarding mouse/human expression differences or “unclear” if this was not the case, indicating that further investigation was necessary.

We found that 50.5% of mRNA differences are validated by matching proteome differences and further 30.3% are compatible. Of those compatible, 15.2% show a clear tendency and for 15.1% the differences are simply not very clear when looking at the proteome data for confirmation of the mRNA differences. Thus, a total of 80.8% of the data show species-specific differences in protein expression in mRNA and protein expression in the same direction. For 19.2% of the data looked at we have clear opposite differences from mRNA expression differences on the protein level.

## Supplementary Information

**Additional file 1:** **The supplemental material.** **Supplemental Table S1.** Proteins and interactions analyzed, all data. **Supplemental Table S2.** Comparison among all data-sets, all results. **Figure S4.** Differences in the central regulatory cascade (CC) between mouse and human, high resolution version for better readability. **Figure S5.** Full Network of proteins in and around the central platelet signalling cascade, high resolution version for better readability.

## Data Availability

All original data of this article, in particular, the protein-protein interaction data as well as the mapping of the gene expression and protein expression data can be found in the article itself and the data supplement available with the online version of this article (Supplemental document, Supplemental excel tables Table S1 and Table S2).
