# Supplementary material for: Comparison of the central human and mouse platelet signaling cascade by systems biological analysis
Source: BMC Genomics. 2020 Dec 22;21:897. doi: 10.1186/s12864-020-07215-4 (PMC7756956; doi:10.1186/s12864-020-07215-4)
Supplement: Supplementary file 1 — Additional file 1: The supplemental material. Supplemental Table S1. Proteins and interactions analyzed, all data. Supplemental Table S2. Comparison among all data-sets, all results. Figure S4. Differences in the central regulatory cascade (CC) between mouse and human, high resolution version for better readability. Figure S5. Full Network of proteins in and around the central platelet signalling cascade, high resolution version for better readability. [file 12864_2020_7215_MOESM1_ESM.zip › SupplementaryDataDocBMCGenomicsF_ESM.docx]

**Supplemental Material**

1. **Full protein networks** of all data analysed:

**Supplemental Figure 1: The flow chart indicates the workflow of the analysis**.

**Supplemental Figure 2: Human network of degree 3 from central cascade indicating the difference between mouse and human.** This network is a subnetwork of **Figure 1** (parameters are the same).

**Supplemental Figure 3: Murine network of degree 3 from central cascade indicating the difference between mouse and human.** This network is a subnetwork of **Figure 1**. Hence, the parameters are the same as in **Figure 1**.

1. **All data on the networks analyzed.** These are given in the attached supplemental excel **Tables S1 und S2**:

**Supplemental Table S1: Proteins and Interactions of the central cascade and its neighbors up to degree 3 in human and murine platelets.** The Table contains detailed data on the proteins and protein interactions. The first register displays all human protein in the network with full gene name, RPKM value, centrality, detection evidence and genomic difference in comparison to mouse. The register nodes in murine network is alike information for mouse. The further two register show proteins unique in the human platelet, as well as proteins unique in the mouse platelet in reciprocal comparison. In addition, the network interactions up to degree 3 from the central cascade and the corresponding prediction scores are given.

**Supplemental Table S2: Dual RNAseq data of mouse and human platelets compared with 11 recent proteomic platelet studies.** Observed protein expression differences in the central signaling cascade of the platelet and their confirmation by different proteomic data-sets. To compare RNAseq data and mass spectrometric data, the RPKM values, spectral counts, signal strength, PSM (peptide spectrum matches), MS/MS counts, protein copy number, or just the binary evidence for detection were analyzed according to the median of the underlying distribution. For each study, the log2-fold change to the basis of the median of the signals was calculated. The latter led to relational abundance values of protein enabling the comparison of several drastically different approaches of measuring protein and mRNA content in platelets. Detailed investigation was done only on interesting mRNA differences mentioned in this paper (99 proteins). Displayed are the proteins, the genomic difference, the RPKM values from Rowley et al., 2011 [1], as well as the binary PlateletWeb[2] evidence as a basis for comparison. In addition, the log2fold change of the proteins according to the respective median of several proteomic studies is stated. If semi-quantitative data were not available only binary information of detection was inserted. The register log2-fold shows solely the log2-fold changes and a color code with a related column that indicates confirmed differences of RNAseq data by the proteomics studies. The color code of log2 changes: blue for high expression and red for low expression. The color code for differences identified by proteomics is green for similar trend in proteomic data and red for different trend of protein abundance than the RNA abundance.

1. **Figure S4. Differences in the central regulatory cascade (CC) between mouse and human:**

The **Supplemental Figure 4** is a separate png file and a high-resolution version of **Figure 1** in the results. It hence has the same legend as the **Figure 1**: The center of the human and murine signaling cascade (defined according to systems biological modelling) and its regulators are presented in a combined network including proteomic, transcriptomic, metabolic and ionic interactors (full data **Figure 2**). In thick edges, the main regulatory interactions are highlighted. The neighbors up to degree 3 are presented (see methods overview for an exact definition of 1^st^ to 3^rd^ degree neighbors of the **CC**. Asterisks label confirmed key expression differences of platelet proteins between human and mouse. As the platelet transcript and validated protein content is around 10181 (9811 protein-coding) in human and 5981 (5814 protein-coding) in mice, large interaction networks can be reconstructed (Human: 18618 high confident interactions and 3524 interactors, Mouse: 10337 high confident interactions and 2114 interactors). In order to outline the important direct and indirect regulators of the central cascade that mark a difference in both species, the combined network shows solely the clear differences (filtered) of a subset of the global interaction network from the first to third neighbors of the central cascade (full: 1811 nodes and 11527 edges; filtered: 411 nodes and 1959 edges). The combined central network separated into species results in 1618 nodes and 9406 edges in human (**Figure S2**), as well as 1061 nodes and 5769 edges in mice (**Figure S3**). The filtered combined central network results in 369 nodes and 1646 edges in human, as well as 277 nodes and 1119 edges in mice. The first to the third neighbor network was filtered according to clear genomic or transcriptomic differences (interspecies expression differences >100 RPKM; expressed >10 RPKM in one species whereas not in the other; no ortholog found between species according to Inparanoid^8^; connector between those proteins). The human and mouse network were combined. The differences in both network topologies are shown in color code. The border paint marks expression values (blue for high expression in human; red for high expression in mouse; grey for no evident expression differences). The node paint marks proteins that occur only in human platelet network (blue), only in human (blue rectangle; non-ortholog proteins), only in murine platelet network (red), only in mouse (red rectangle; non-ortholog proteins), or in both (white). The grey fill color of nodes indicates proteins that are not expressed in platelets in either species. Second messengers (e.g. Calcium, ATP, ADP) are also shown in grey. The node size increases with high expression differences. Edge colors indicate interactions in both species (grey), in human (blue), in mouse (red) and in the central cascade (dark grey). Selected high protein expression differences which are shown by transcriptomics and proteomics accordingly (**Table 1** and **Figure 3**) are highlighted by golden asterisks. High binders above 90% percentile were excluded. Abbreviations in the figure are the Entrez gene symbols and the full names are given for all genes in **Supplemental Table 1**.

1. **Figure S5. Full network of the differences in the central regulatory cascade (CC) between mouse and human:**

The **Supplemental Figure 5** is a separate png file and a high-resolution version of **Figure 2** in the results. It hence has the same legend as the **Figure 2**: Full Network of proteins in and around the central platelet signaling cascade. The human and mouse networks were combined. The differences in both network topologies are shown in color code. The border paint marks expression values (blue for high expression in human; red for high expression in mouse; grey for no considerable expression differences). The node paint marks proteins that occur only in human platelet network (blue), only in human (blue rectangle; non-ortholog proteins), only in murine platelet network (red), only in mouse (red rectangle; non-ortholog proteins), or in both (white). The grey fill color of nodes indicates proteins that are not expressed in platelets in both species, or second messenger (e.g. Calcium, ATP, ADP). The node size increases with high expression differences. Further, edge color indicates interactions in both species (grey), in human (blue), in mouse (red) and in the central cascade (dark grey). High binders above 90% percentile where excluded. Abbreviations in the figure are the Entrez gene symbols and the full names are given for all proteins in **Supplemental Table 1**.

1. **Detailed discussion of individual differences in protein expression between mouse and human platelets** (extended text discussion of the found differences)


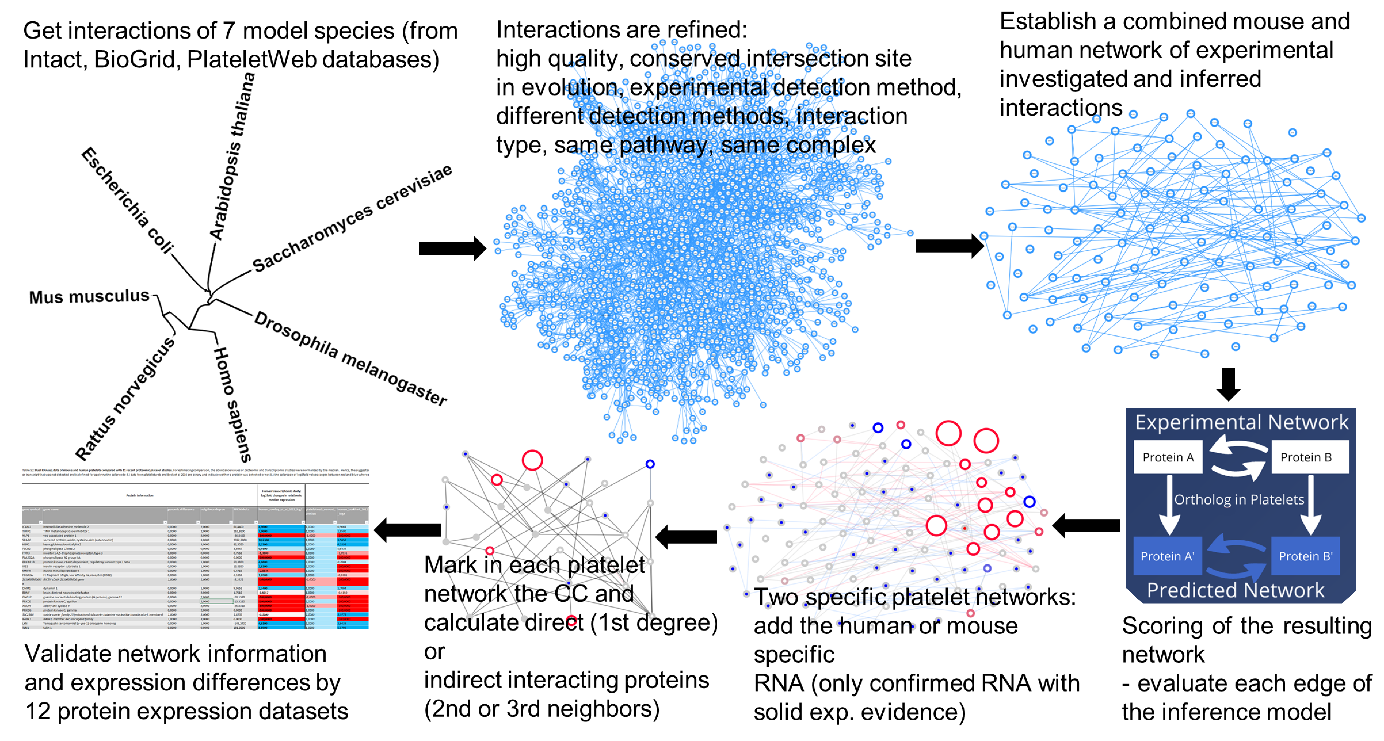


**Figure S1: Workflow Chart**. For reconstructing the platelet network 3 different sources were used (IntAct, Biogrid, PlateletWeb). As the network coverage is heterogenic for mouse and men in the experimental databases, we decided to combine the experimental interactions with an inference method. For this the protein interactions of 7 species were combined to an overall network. Only high quality interactions were used. To improve the quality, the interactions were filtered by its evolutionary conservation of the intersection, the detection method, the number of different detection methods, the same pathway and complex occurrence, as well as the interaction type. Based on the multispecies network, a homogenic interaction network in mouse and human was deduced. The interactions of that network were scored and high scoring interactions were selected for final analysis. The platelet network is derived by combining the human and mouse network with transcriptomics. Further, previous network analysis already outlined the central pathway of platelet regulation. This module, the central cascade (**CC**), was used as additional filter, for outlining evident differences between mouse and men in platelet. In a final step, 12 proteomic studies (11 MS studies + PlateletWeb) were used to evaluate the transcriptomic differences. The expression differences that were consistent over the different datasets were highlighted in Table1 and Figure 3 and are suggested for further analysis. The phylogentic tree was constructed by iTOL [3]. The central regulating cascade of platelet activation (**CC**) was defined according to systems biological modelling, directly **CC** interacting proteins were defined as 1^st^ degree neighbors, their interactor are 2^nd^ degree neighbors of the **CC** and finally, the neighbors of these 2^nd^ degree neighbors are 3^rd^ degree neighbors of the **CC**.

**
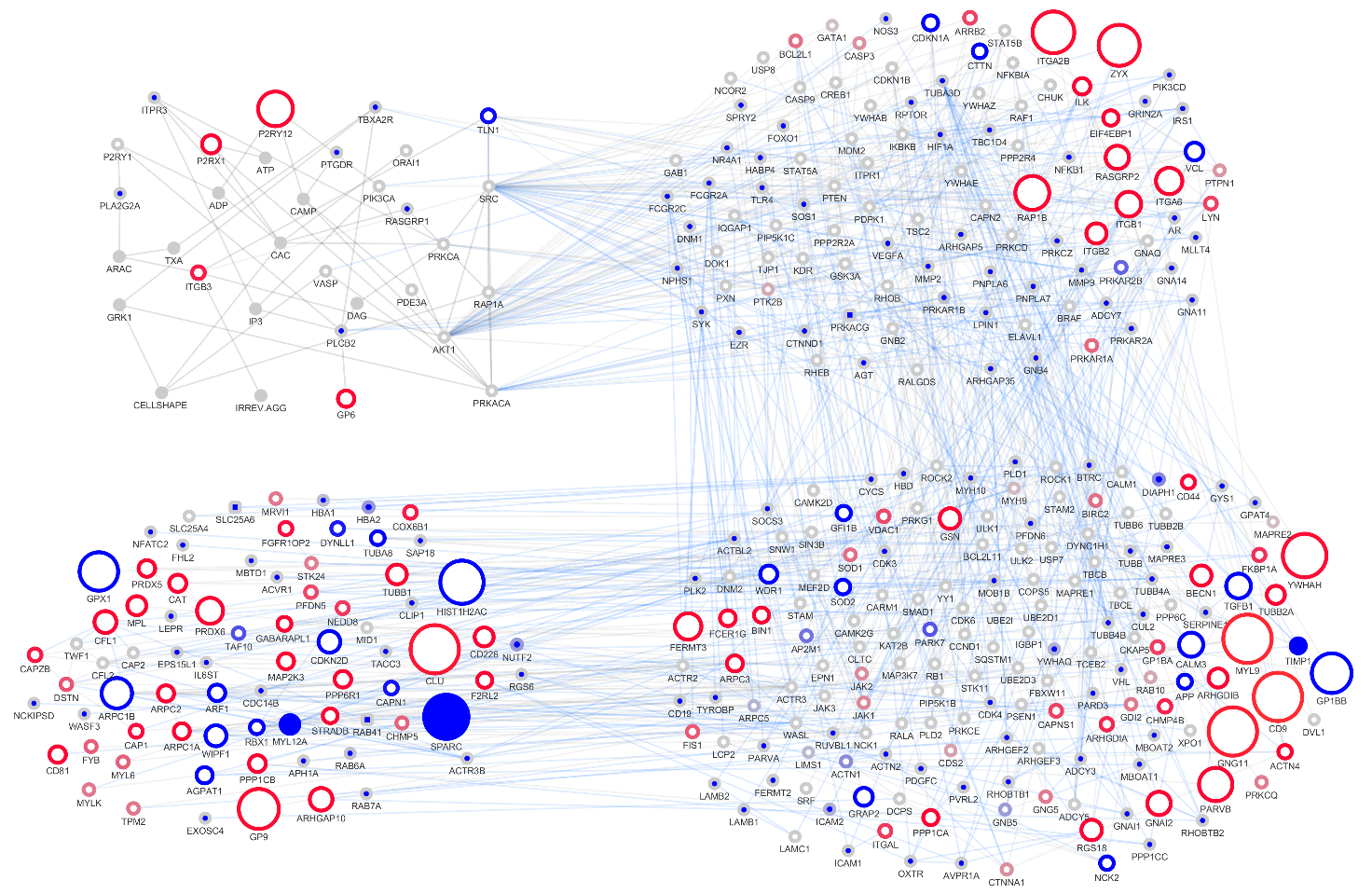
**

**Figure S2: Human only network of the central platelet signaling cascade.** Edges and nodes that solely exist in mouse were excluded. Major differences according to Figure 1 are presented. The differences in both network topologies are shown in color code. The border paint marks expression values (blue for high expression in human; red for high expression in mouse; grey for no considerable expression differences). The node paint marks proteins that occur only in human platelet network (blue), only in human (blue rectangle; non-ortholog proteins), only in murine platelet network (red), only in mouse (red rectangle; non-ortholog proteins), or in both (white). The grey fill color of nodes indicates proteins that are not expressed in platelets in both species, or second messenger (e.g. Calcium, ATP, ADP). The node size increases with high expression differences. Further, edge color indicates interactions in both species (grey), in human (blue), in mouse (red) and in the central cascade (dark grey). High binders above 90% percentile where excluded. Abbreviations in the figure are the Entrez gene symbols and the full names are given for all proteins in **Supplemental Table 1**.

**
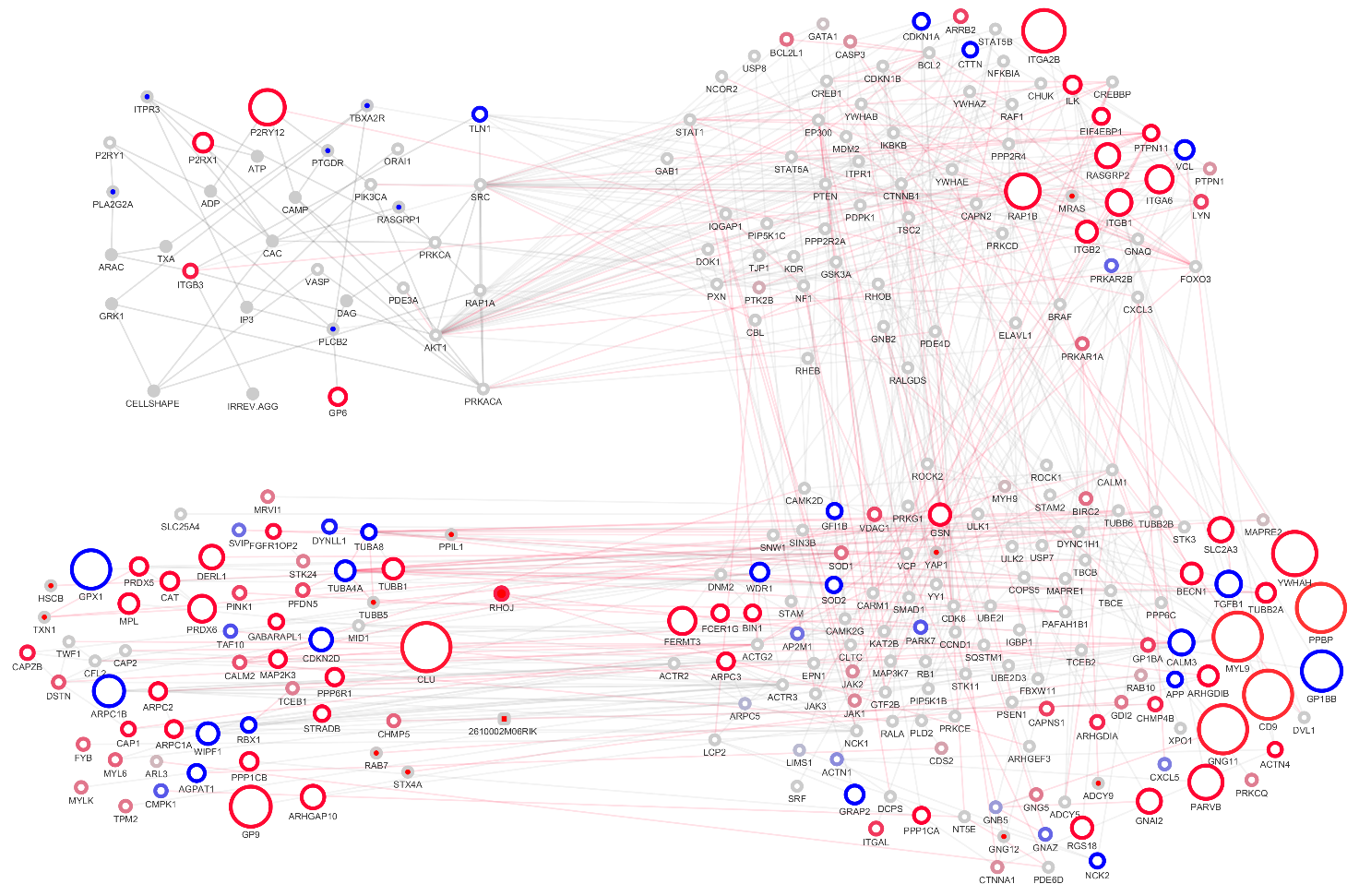
**

**Figure S3: Mouse only network the central platelet signaling cascade.** Edges and nodes that solely exist in human were excluded. Major differences according to Figure 1 are presented. The differences in both network topologies are shown in color code. The border paint marks expression values (blue for high expression in human; red for high expression in mouse; grey for no considerable expression differences). The node paint marks proteins that occur only in human platelet network (blue), only in human (blue rectangle; non-ortholog proteins), only in murine platelet network (red), only in mouse (red rectangle; non-ortholog proteins), or in both (white). The grey fill color of nodes indicates proteins that are not expressed in platelets in both species, or second messenger (e.g. Calcium, ATP, ADP). The node size increases with high expression differences. Further, edge color indicates interactions in both species (grey), in human (blue), in mouse (red) and in the central cascade (dark grey). High binders above 90% percentile where excluded. Abbreviations in the figure are the Entrez gene symbols and the full names are given for all proteins in **Supplemental Table 1**.

**Individual differences in protein expression between mouse and human central signaling cascade**

Data on genomic, gene expression and proteome differences are summarized in Figure 1. Sole expression in one species was noted. Hub proteins above the 90% percentile regarding the number of interactions were excluded. All differences observed after a first screening of genome and transcriptome were next evaluated comparing different data sets (**Table S2**), as well as hand-curated in view of available literature. Also unresolved annotation differences were noted, for instance regarding the protein clusterin (CLU): There is a non-coding transcript variant of CLU occurring in human.

SOD2 (Super oxide dismutase 2) is higher abundant in human and pivotal for redox protection and responses in platelet thrombo-inflammation (**Table S1**). Conversely, SOD1 (Super oxide dismutase 1) is decreased in human. Both differences are confirmed by several proteomic studies (**Table S2**). In general, SOD (superoxide dismutase) enzymes control the expression levels of a variety of reactive oxygen species (ROS) in the mitochondria, limiting the potential toxicity of ROS thereby controlling signaling functions [4]. In line with this, genetic deletion of SOD2 in mouse platelets increases the level of mitochondrial ROS, but in sharp contrast, total platelet ROS production was unchanged. Although decreased mitochondrial function following thrombin stimulation was observed in SOD2-deficient platelets, this defect does not influence platelet activation, spreading or arterial thrombosis or hemostasis in vivo. Interestingly, we found that expression levels of SOD1 and 2 are different between mouse and human. In mouse, SOD1 is highly expressed whereas in contrast, SOD2 is dominant isoform in human and SOD1 expression is much less than observed in mouse. Altogether, our results suggest that SOD2 functions seem to be redundant in mouse, SOD1 isoforms may partially compensate the lack of SOD2 function in mouse platelets [5].

Moreover, mRNA expression of TLN1 (Talin 1) was higher in human, compared to mouse, in accordance with proteomic studies. In contrast, mRNA expression of TLN2 (Talin 2) was not detected neither in human nor murine platelets, but high protein expression levels were found in both species. Altogether, our findings indicate that the observed differences between mouse and human central cascades clearly affect platelet reactivity. Such expression-sensitive behavior of platelet reactivity has been described earlier for the Src activating switch [6]. However, their combined effect on platelet reactivity is usually not taken into account.

Purinergic receptor signalling is regulated by P2Y1/P2Y12 receptors and calcium channel P2X1 which are directly activated by ADP and ATP, respectively [7, 8]. ADP and ATP are stored in platelet δ-granules and released upon platelet activation. Antiplatelet drugs such as Clopidogrel, Prasugrel and Ticagrelor have been frequently used to treat patients with cardiovascular diseases thereby inhibiting ADP-induced platelet activation and avoid uncontrolled thrombus formation. Increase of calcium levels through P2X1 is also contributed to enhance inside-out activation αIIbβ3 integrin activation, which leads to platelet adhesion and aggregation. Interestingly, we could detect higher expression levels of P2Y1/P2Y12 and P2X1 in mouse compared to human, but the protein is higher abundant in human. Genetic deletion of P2X1 strongly inhibits collagen-mediated thrombus formation in vivo [2, 9]. In line with that GPVI and higher expression in mice was found but protein level I is higher in men. Integrin ß3 shows very small difference between human and mouse in transcriptome and proteome.

Platelets express three collagen receptors: glycoprotein VI (GPVI), glycoprotein V (GPV) and integrin α2β1. The platelet receptor for collagen, laminin and fibrin, GPVI is a 58-60 kDa type I transmembrane protein, that centrally regulates multiple platelet functions, including adhesion, activation, aggregation and pro-coagulant activity [1, 3-6, 10]. Structurally, GPVI contains two IgG domains with sites for collagen binding. The cytoplasmic tail forms the binding site for Src-family tyrosine kinases, protein kinase C and calmodulin. GPVI signaling also requires the binding to immunoreceptor tyrosine-based activation motif (ITAM)-bearing IgG Fc receptor FcR γ-chain, which is non-covalently associated with the cytoplasmic tail of GPVI [7]. Collagen binding to the extracellular domain of GPVI induces dimerization of the receptor, causing a conformational change that brings together the cytoplasmic tails and ITAMs, facilitating phosphorylation of ITAMs, thereby initiating intracellular signals, thus resembling the GPVI signalosome [8]. It has been shown that GPVI dimers also form collagen-mediated clusters and are associated to integrins. Therefore, low copy number of GPVI represents only limited number of binding sites on collagen thereby attenuating collagen-dependent receptor clustering. We observed that GPVI and integrin densities on the mouse platelet surface are contrasting human, suggesting that GPVI/integrin clustering may be different between mouse and human.

RasGRP2 (RAS guanyl-releasing protein 2) was found to be considerably higher expressed in murine platelets. PLCB2 (1-Phosphatidylinositol-4,5-bisphosphate phosphodiesterase beta-2) appears to be only present in human (**Table S2**). Here it will affect secondary messenger levels of phosphatidyl inositols. In addition, the five interactors SLC25A6, BDNF, PRKAR1B, DNM1, IRS1, MMP9, FCGR2A and ITPR3 were only found in human, but not detected in mouse.

ITPR3 (inositol 1,4,5-trisphosphate receptor, type 3) is directly connected in the central cascade interacting with cytosolic calcium and Inositoltrisphosphat. PRKAR1B (protein kinase, cAMP-dependent, regulatory subunit type I beta), IRS1 (insulin receptor substrate 1), MMP9 (matrix metallopeptidase 9), FCGR2A (Fc fragment of IgG, low affinity IIa, receptor (CD32)) and DNM1 (dynamin 1) are 1st degree neighbors.

Finally, GNG12 (guanine nucleotide binding protein (G protein), gamma 12), PRKCE (protein kinase C, epsilon) and ADCY9 (adenylate cyclase 9) are highly abundant, which is also valid for proteomics and only found in mouse. All are second degree neighbors,

BDNF (brain derived neurotrophic factor) is a secretory protein regulating the development and function of neural circuits. BDNF and platelet dysfunction is associated with the pathophysiology of depressive disorders [11]. BDNF is stored in α-granules of human platelets [9]. We show that BDNF is a 2nd degree neighbor in our network (mRNA and protein detection), and gene transcript and protein is not present in mouse platelets. Using in vitro grown human and mouse megakaryocytes, comprehensive analyses convincingly showed that BDNF mRNA is only expressed in human megakaryocyte/platelet lineage. Because, BDNF is a 2nd degree neighbor in our network, we expected to have definite influence on platelet function, whether any other neutrophin isoform (NT3, 4 or 5) or NGF may compensate BDNF function in mouse platelets. However, none of these proteins is present in the mouse central network and NGF is neither detected in platelet transcriptome nor in proteome of both species. In contrary, many isoform of NT5 (NT5C (only human), NT5C2, NT5C3, NT5DC1-2, NT5E (only mouse), NT5M exists in the platelet transcriptome. Therefore our results also suggests that none of the nerve growth factor family members is able to compensate the lack of BDNF function in murine platelets [12], similarly as earlier described [12].

For our approach we selected the central activating cascade of platelet thrombosis and hemostasis, however, further transcriptome comparisons are also revealing clear differences between murine and human platelets.

For the interactions around the central cascade, we could observe clear differences. For example, metalloproteinase inhibitor 1 (TIMP1) is not expressed in mice, but on the other hand, TIMP1 has the highest expression level of all metalloproteinase-associated proteins in humans. Likewise, the protein level is again high in human, whereas the protein shows very low abundance in mouse in contrary to the not detected transcript. This could have effect on the underlying regulatory circuits in human, which are not present in mouse. We also found that matrix metalloproteinase 9 (MMP9) had unique but low expression levels in human, comparing to mouse. Metalloproteinase inhibitor TIMP3 is only expressed in mouse but at low level. On protein level metalloproteinase inhibitor 3 (TIMP3) appears in both organism but higher in mice. TIMP2 shows low expression in both species. In contrast, metalloprotease ADAM17 has low level but is increased in mouse. On protein level the difference is more distinct.

We also showed that the number of cytoskeleton-associated integrins or integrin-linked kinases in human was higher (18 proteins) than in mouse (13 proteins). However, the expression value of integrins in mice is higher. The sum of RPKM delta of integrins present in both species is -26151.9 (13 proteins). Putatively, the higher expression values within the integrin group in mouse are compensated for by specialization in human. Similar to this in the human network there are 14 guanine nucleotide-binding proteins (GNA11, GNA12, GNA13, GNA14, GNAI1, GNAI2, GNAI3, GNAL, GNAO1, GNAQ, GNAT1, GNAT2, GNAT3, GNAZ) and in the murine network there are only 6 of them (GNA12, GNA13, GNAI2, GNAI3, GNAQ, GNAZ), again with drastically increased abundance in mouse, ( e.g. RPKIM delta of -567.8 regarding GNAI2).

The tetraspanin CD9 (second neighbor) has a very high expression level only in mice (RPKM mouse: 7081; RPKM human: 153.2), which is confirmed by proteomics. The fourth neighbors CD226 or CD81 are also highly expressed in mouse, CDKN2D (third neighbor) has a RPKM of 510.1 in human and 3.9 in mice.

**Major differences in expression**

For several interactions in the **CC** as well as around it (up to 3^rd^ degree neighbors) we could observe clear differences. For example, **TIMP1** is barely expressed in mouse platelets, but on the other hand, TIMP1 has the highest expression level of all metalloproteinase-associated proteins in human platelets. The RNAseq results are matching with proteomics. This could have an effect on the underlying regulatory circuits in both species. We also found that matrix metalloproteinase **MMP9** had unique but low expression levels in human, compared to mouse (results are matching proteomics). Metalloproteinase inhibitor **TIMP3** is only highly abundant in mouse (matches proteomics), whereas **TIMP2** shows low protein expression in both species with the tendency of higher expression in human (matches proteomics). In contrast, metalloprotease **ADAM17** is more abundant in mouse (matches proteomics). In addition, we also found that super oxide dismutase 2 (**SOD2**) is considerably more abundant in human than in mouse platelets. Conversely, **SOD1** is highly expressed in platelets but has a lower abundance in human compared to mouse. Both differences are matching with proteomic studies (**Table S2**).

**PRKAR1B** (protein kinase, cAMP-dependent, regulatory subunit type I beta) is highly expressed in human, but not detected in mouse on proteome and transcriptome level, thus marking a major difference between mouse and human platelets.

**BDNF** and **ITPR3** are present in both genomes but absent in all murine transcriptome and proteome datasets yet detected in human transcriptome and proteome. The mouse has unique **CC** modulating proteins **GNG12**, **PRKCE** and **ADCY9**. **CALM3** is higher expressed in human platelets, whereas **ITGB2** and **MYL9** have higher expression levels in mouse platelets. These results are valid on transcriptome and proteome levels.

**Further discussion of found expression differences:** This concerns higher expression levels in human platelets, e.g.**TLN1** (talin 1), **CALM3** (calmodulin 3), **PRKCB** (protein kinase C, beta), **APP** (amyloid beta (A4) precursor protein), **TIMP1** (tissue inhibitor of metalloproteinase 1) and **SOD2** (superoxide dismutase 2). Important and clear differences involve **PLCB2** (phospholipase C beta 2; central), **BDNF (**brain-derived neurotrophic factor; degree 2), **MMP9** (matrix metallopeptidase 9; degree 1), **ITPR3** (inositol 1,4,5-trisphosphate receptor, type 3; central), and **SLC25A6** (matrix metallopeptidase 9; degree 3) which have only been detected in human platelets. In contrast, higher expressed in mice are **ADAM17** (ADAM metallopeptidase domain 17), **SOD1** (superoxide dismutase 1, soluble), **RASGRP2** (RAS, guanyl releasing protein 2**),** **ITGB3** (integrin beta 3), **MYL9** (myosin, light polypeptide 9, regulatory), **EIF4EBP1** (eukaryotic translation initiation factor 4E binding protein 1), **ARRB2 (**arrestin, beta 2), **CD9** (CD9 antigen) and **TIMP3** (TIMP metallopeptidase inhibitor 3**)**.

Moreover, human platelets lack regulation by **GNG12** (guanine nucleotide-binding protein (G protein), gamma 12; degree 2**)**, **PRKCE** (protein kinase C, epsilon; degree 2) and **ADCY9** (adenylate cyclase 9; degree 2).

The latter differences are again highlighted as *clear*, as those mark binary differences of a protein measured in one species but not in the other. The actual effects on the dynamics of platelet responses require further analysis as isoforms can replace missing proteins; in addition, there are feedback loops and higher order effects and limits of MS sensitivity [11].

In the **CC** of mouse and human platelets, we made the following observations: the central surface receptors **P2RY12** (purinergic receptor P2Y, G-protein coupled, 12) has a higher mRNA expression in mice. On protein level, P2RY12 is easily quantified in mice. However, due to its low quantity in human platelets, it is more challenging to measure. There are low but variable expression levels of P2RY12 protein [9]. Our results imply a higher abundance of P2RY12 in mice. The transcriptome data are compatible with the proteome data. Regarding the receptor P2RX1 transcriptomics indicates higher mRNA expression levels in mice, but proteomics show higher abundance values for human. This would suggest translational expression differences but further data are necessary to be sure. Furthermore, ITGB3 (integrin beta 3) showed a strongly increased level of mRNA in human, and also on protein level it is higher. For GP6 (glycoprotein VI (platelet) the mRNA level tends to be increased in mice, but the protein level shows clearly higher abundance in human. In addition the **SRC** kinase in **CC,** shows interesting abundance differences, higher protein level in humans, but higher mRNA levels in mouse. As mentioned above, clear and matched differences in CC (mRNA expression differences that are clearly supported by protein abundancies; see supplement) are found regarding **PLCB2**, 1-Phosphatidylinositol-4,5-bisphosphate phosphodiesterase beta-2, which appears to be only present in human where it affects second messenger levels, such as diacylglycerol (**Table S1**).

**TLN1** (Talin) is more abundant in human and **ITPR3** occurs only in human. Talin is important for cytoskeletal linkage of the CC and loss of Talin leads to defective integrin αIIbβ3-mediated platelet aggregation and β1 integrin–mediated platelet adhesion [13]. ITPR denotes the Inositol 1,4,5-Trisphosphate Receptor Type 3. It has a calcium channel (C-terminus) and a ligand-binding site (N-terminus).

**Specific genomic differences in the 1^st^ to 3^rd^ degree neighbors**

Regarding genomic differences of 1^st^ to 3^rd^ degree neighbors of the central cascade we identified 5 human genes not detected in the mouse genome: **SLC25A6** (solute carrier family 25, member A6), CASP10 (caspase 10), PRKACG (protein kinase, cAMP-dependent, gamma catalytic subunit; the only human-specific 1^st^ degree neighbor), **HSPA6** (heat shock protein family A (Hsp70) member 6) and RAB41 (RAB41, member RAS oncogene family). HSPA6 and SLC25A6 are highly abundant on protein level (**Table S2**). However, PRKACG and CASP10 show transcript evidence but no protein evidence.

On the other hand, two mouse-specific genes were found in genome databases (TLR13, toll-like receptor 13 and **2610002M06RIK**, RIKEN cDNA 2610002M06 gene), which do not exist in human. Only 2610002M06RIK was detected in the mouse platelet transcriptome and proteome. 2610002M06RIK is a direct interactor of the central cascade and thus might influence signaling. In contrary, TLR13 is only detected in transcriptome.

**Further details on different expression profile of the 2^nd^ to 3^rd^ degree neighbors:**

Regarding the **2^nd^ neighbors** (543 human mRNAs) we identified 134 proteins in human which were not found in mouse platelets. Some of these proteins were exclusively and highly expressed in human platelets (see methods) such as **TIMP1** (log2: 7.1 for mRNA and 2.6 for protein), DIAPH1 (compatible), ICAM2 (transcriptomics are supported by proteomics) and YWHAQ (proteomics support). The higher abundance of TIMP1 mRNA matches with higher protein abundance of TIMP1 in human and there is no evidence for mRNA, or protein in mice. In contrast to the absence of mRNA detection in mouse, protein evidence of DIAPH1 (log2: 3.8, Zeiler et al, 2014 [14]), and YWHAQ (log2: 4.7 Zeiler et al, 2014 [15]) was found in both species. However, human protein abundance is still higher.

There were 32 mRNAs identified only in mouse platelets (of 365 murine 2^nd^ neighbours), which were not detected in human platelets. According to the relative expression profile, most dominant are ARPC3 (high protein level in both species), YAP1 (matching protein data), TUBB2B (matching; high mouse protein level; log2: 5.0), ADCY9 (matching; log2 protein: 3.1), GNG12 (matching; log2 protein: 3.8) and PRKCE (matching; log2 protein: 2.3).

13 ortholog mRNAs are expressed in both species, but are more abundant in human. The expression levels of CALM3, SOD2, APP and PRKCB are in accordance with proteome data.

However, there are 46 direct ortholog mRNAs that are clearly higher abundant in mice though also detected in human. The increased abundance in mice is also found on protein level for, e.g. CD9, MYL9, GNG11, YWHAH, PARVB, SLC2A3 and GNAI2 (further information in **Table** **S1**). However, six human proteins, all 2nd-degree neighbors of the central cascade (GP1BB, TGFB1, WDR1, NCK, AP2M1, CXCL5 (chemokine (C-X-C motif) ligand 5) showed higher corresponding mRNA expression in human platelets, but higher protein levels are detected in mouse platelets.

Referring to mRNA levels, in the **3^rd^ degree neighbor** network, 285 mRNAs are only detected in human, but not in mouse platelets, whereas 58 mRNAs are exclusively detected in mouse platelets. 24 of the 3^rd^ neighbor mRNAs show a stronger expression in human. 46 platelet mRNAs are stronger expressed in mice, e.g. CLU and RHOJ. In the following, interesting differences are highlighted. CLU was also detected highly abundant in the human platelet proteome and on a low level in the mouse proteome. RHOJ is not detected in the proteome of either species. ADAM17 (log2 human protein: 2.2) is more abundant in mouse mRNA and protein. In contrast, SPARC (matching tendency and high difference), MYL12A (matching tendency) and HBA2 (matching tendency and high difference) were only and highly detected in human, but not in mouse platelets.

Comparing the expression profile of a total of 1811 platelet proteins, we could detect 243 proteins which were differentially expressed between mouse and human, or solely represented in one of the two species. Interesting differences were defined by a genomic difference, or a delta RPKM difference of more than 100, or mRNAs that are only detected in one species.

**Expression levels that point to opposite directions if comparing proteome expression data with RNA expression data**

It is important to note that in 19% of the cases the datasets between proteome and RNA expression data point to opposite directions regarding higher expression in mouse or human (**Table S2)**, requiring further experimental investigation. Such opposite levels of abundance differences concern 4 protein of the central cascade (SRC, TBXA2R, PTGDR, RASGRP1).

**Table S2** shows in addition that there are 8 out of 37 for 1^st^ neighbor differences in expression clearly opposing proteome (e.g. SYK; PRKAR2B, protein kinase, cAMP-dependent, regulatory subunit type II beta; CTTN, cortactin; NF1, neurofibromin 1). Finally, only 4 out of 38 of the 2^nd^ degree neighbors have such differences (CXCL5, chemokine C-X-C motif ligand 5; GP1BB, glycoprotein Ib, beta polypeptide; GFI1B; PRK7; **Table S2**). The combined network of the central platelet signaling cascade compares and combines human and murine interactions. These cover together 1801 proteins from both species with 1743 human and 1180 murine proteins. (**Figure 2 and Table S1**).

**References**

1. Versteeg HH, Heemskerk JW, Levi M, Reitsma PH: **New fundamentals in hemostasis.** *Physiol Rev* 2013, **93:**327-358.

2. De Meyer SF, Denorme F, Langhauser F, Geuss E, Fluri F, Kleinschnitz C: **Thromboinflammation in Stroke Brain Damage.** *Stroke* 2016, **47:**1165-1172.

3. Kraft P, Nieswandt B, Stoll G, Kleinschnitz C: **[Acute ischemic stroke. New approaches to antithrombotic treatment].** *Nervenarzt* 2012, **83:**435-449.

4. Bertozzi CC, Hess PR, Kahn ML: **Platelets Covert Regulators of Lymphatic Development.** *Arteriosclerosis Thrombosis and Vascular Biology* 2010, **30:**2368-2371.

5. Bertozzi CC, Schmaier AA, Mericko P, Hess PR, Zou ZY, Chen M, Chen CY, Xu B, Lu MM, Zhou D, et al: **Platelets regulate lymphatic vascular development through CLEC-2-SLP-76 signaling.** *Blood* 2010, **116:**661-670.

6. Mammadova-Bach E, Freund M, Bagnard D, Gachet C, Mangin P: **Platelet integrin alpha 6 beta 1 promotes metastatic dissemination.** *Journal of Thrombosis and Haemostasis* 2015, **13:**34-34.

7. Nurden AT: **Platelets, inflammation and tissue regeneration.** *Thrombosis and Haemostasis* 2011, **105:**S13-S33.

8. Semple JW, Italiano JE, Freedman J: **Platelets and the immune continuum.** *Nature Reviews Immunology* 2011, **11:**264-274.

9. Mischnik M, Boyanova D, Hubertus K, Geiger J, Philippi N, Dittrich M, Wangorsch G, Timmer J, Dandekar T: **A Boolean view separates platelet activatory and inhibitory signalling as verified by phosphorylation monitoring including threshold behaviour and integrin modulation.** *Molecular Biosystems* 2013, **9:**1326-1339.

10. Mammadova-Bach E, Mangin P, Lanza F, Gachet C: **Platelets in cancer From basic research to therapeutic implications.** *Hamostaseologie* 2015, **35:**325-336.

11. Mischnik M, Gambaryan S, Subramanian H, Geiger J, Schutz C, Timmer J, Dandekar T: **A comparative analysis of the bistability switch for platelet aggregation by logic ODE based dynamical modeling.** *Molecular Biosystems* 2014, **10:**2082-2089.

12. Mischnik M, Hubertus K, Geiger J, Dandekar T, Timmer J: **Dynamical modelling of prostaglandin signalling in platelets reveals individual receptor contributions and feedback properties.** *Molecular Biosystems* 2013, **9:**2520-2529.

13. Stegner D: **SePARating thrombosis and hemostasis?** *Thrombosis Research* 2016, **145:**140-142.

14. Schuhmann MK, Guthmann J, Stoll G, Nieswandt B, Kraft P, Kleinschnitz C: **Blocking of platelet glycoprotein receptor Ib reduces "thrombo-inflammation" in mice with acute ischemic stroke.** *Journal of Neuroinflammation* 2017, **14**.

15. Deppermann C, Kraft P, Volz J, Schuhmann MK, Beck S, Wolf K, Stegner D, Stoll G, Nieswandt B: **Platelet secretion is crucial to prevent bleeding in the ischemic brain but not in the inflamed skin or lung in mice.** *Blood* 2017, **129:**1702-1706.
