# Supplementary figures and images for: Comparison of the central human and mouse platelet signaling cascade by systems biological analysis
Source: BMC Genomics. 2020 Dec 22;21:897. doi: 10.1186/s12864-020-07215-4 (PMC7756956; doi:10.1186/s12864-020-07215-4)

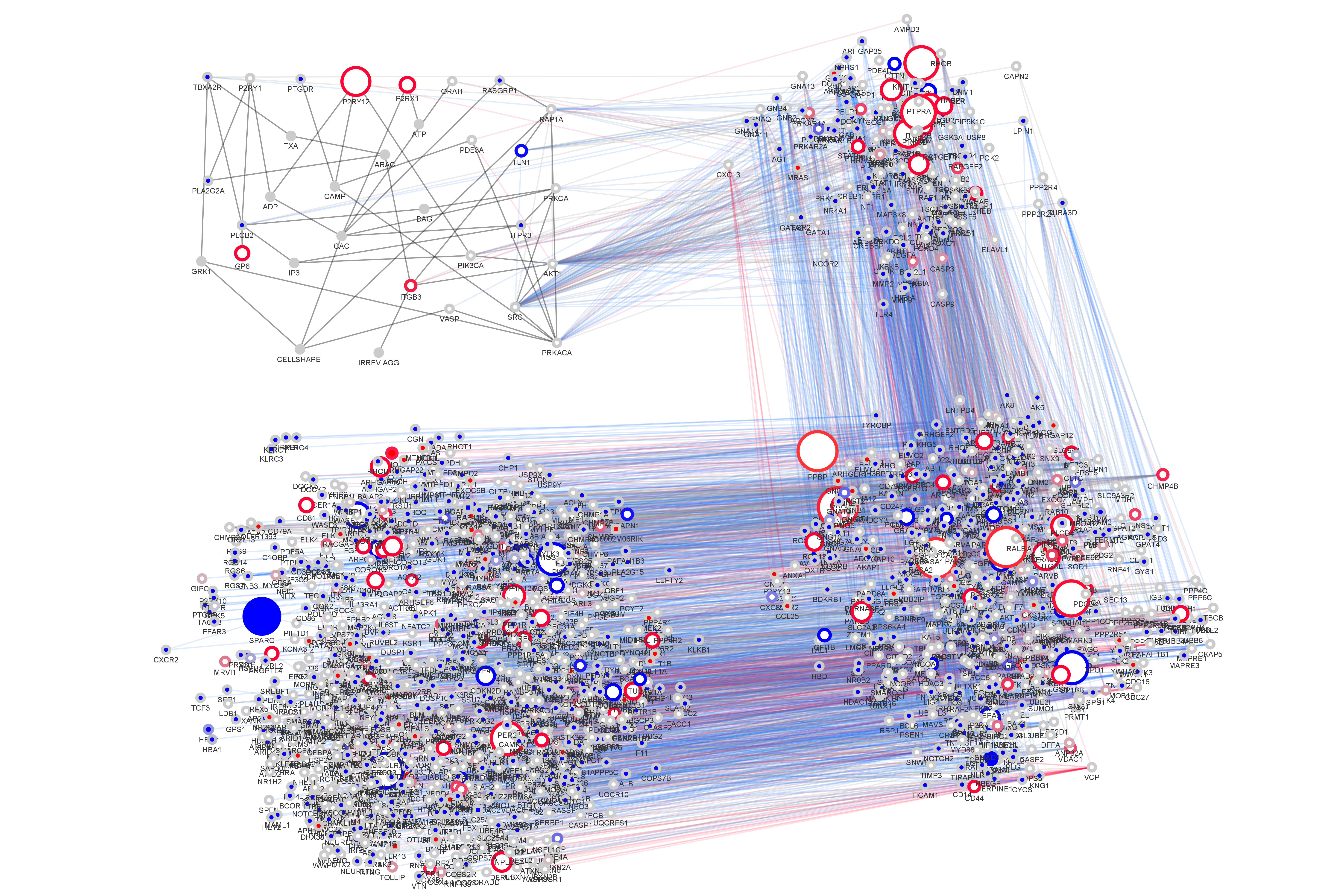

Supplement: Supplementary file 1 — Additional file 1: The supplemental material. Supplemental Table S1. Proteins and interactions analyzed, all data. Supplemental Table S2. Comparison among all data-sets, all results. Figure S4. Differences in the central regulatory cascade (CC) between mouse and human, high resolution version for better readability. Figure S5. Full Network of proteins in and around the central platelet signalling cascade, high resolution version for better readability. [file 12864_2020_7215_MOESM1_ESM.zip › FigS5FullNetworkHQ_ESM.png]

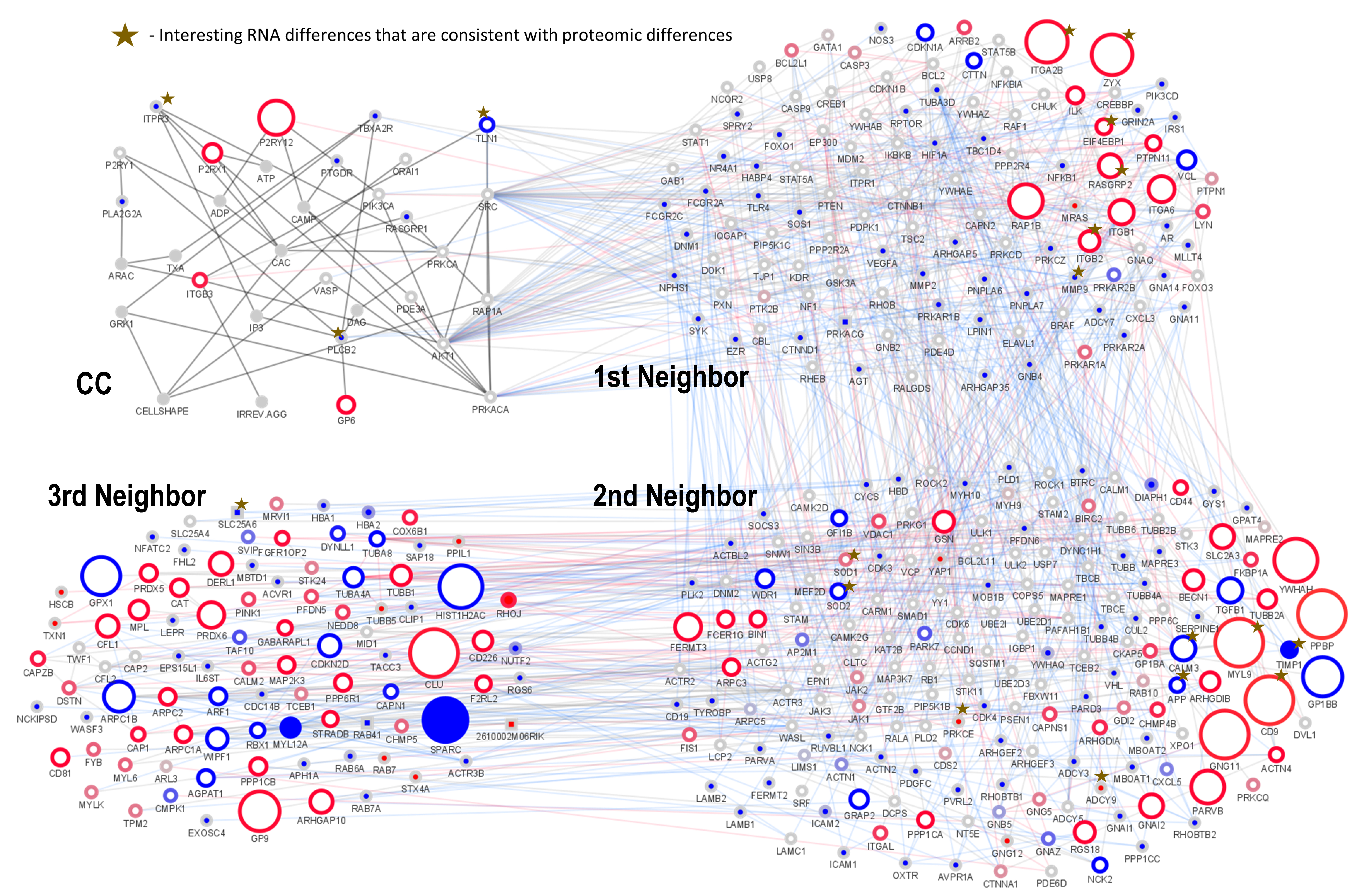

Supplement: Supplementary file 1 — Additional file 1: The supplemental material. Supplemental Table S1. Proteins and interactions analyzed, all data. Supplemental Table S2. Comparison among all data-sets, all results. Figure S4. Differences in the central regulatory cascade (CC) between mouse and human, high resolution version for better readability. Figure S5. Full Network of proteins in and around the central platelet signalling cascade, high resolution version for better readability. [file 12864_2020_7215_MOESM1_ESM.zip › FigS4CCsubnets_ESM.jpg]
